# Analysis of triacylglycerol and phospholipid *sn‐*positional isomers by liquid chromatographic and mass spectrometric methodologies

**DOI:** 10.1002/mas.21853

**Published:** 2023-06-06

**Authors:** Mikael Fabritius, Baoru Yang

**Affiliations:** ^1^ Food Sciences, Department of Life Technologies University of Turku Turku Finland; ^2^ Shanxi Center for Testing of Functional Agro‐Products Shanxi Agricultural University Taiyuan China

**Keywords:** liquid chromatography, mass spectrometry, phospholipid, regioisomer, triacylglycerol

## Abstract

Analysis of triacylglycerol (TG) and phospholipid *sn‐*positional isomers can be divided into two main categories: (a) direct separation by chromatography or other means such as ion mobility mass spectrometry and (b) quantification of regioisomer ratios by structurally informative fragment ions with mass spectrometric methods. Due to long retention times and limited performance, researchers are moving away from direct chromatographic separation of isomers, using mass spectrometry instead. Many established analytical methods are targeting specific isomers of interest instead of untargeted analysis of comprehensive profiles of regioisomers. Challenges remain arising from the large number of isobaric and isomeric lipid species in natural samples, often overlapping chromatographically and sharing structurally informative fragment ions. Further, fragmentation of glycerolipids is influenced by the nature of the attached fatty acids, and the lack of available regiopure standards is still an obstacle for establishing calibration curves required for accurate quantification of regioisomers. Additionally, throughput of many methods is still quite limited. Optimization algorithms and fragmentation models are useful especially for analysis of TG regioisomers, as identification using calibration curves alone without proper separation is difficult with complex samples.

**Abbreviations**
ACN: acyl carbon number
ACN:DB: acyl carbon number: double bond number
Ag‐HPLC: silver ion chromatography
APCI: atmospheric pressure chemical ionization
APPI: atmospheric pressure photoionization
CCS: collisional cross section
CI: chemical ionization
CID: collision‐induced dissociation
DB: double bond
DESI: desorption electrospray ionization
DG: diacylglycerol
DMS: differential mobility spectrometry
DTIMS: drift tube ion mobility spectrometry
ECN: equivalent carbon number
EID: electron‐induced dissociation
EIEIO: electron impact excitation of ions from organics
ESI: electrospray ionization
FA: fatty acid
HCD: higher‐energy collisional dissociation
HESI: heated electrospray ionization
HILIC: hydrophilic interaction liquid chromatography
IMS: ion mobility spectrometry
LC: liquid chromatography
MALDI: matrix‐assisted laser desorption/ionization
MS: mass spectrometry
MS^2^
: tandem mass spectrometry
MS^n^
: sequential fragmentation with *n* product ion stages
nanoESI: nanoelectrospray ionization
OzID: ozone‐induced dissociation
PA: phosphatidic acid
PB: Paterno‐Buchi photochemical reaction
PC: phosphatidylcholine
PE: phosphatidylethanolamine
PG: phosphatidylglycerol
PI: phosphatidylinositol
PL: phospholipid
PS: phosphatidylserine
*rac*
: racemic
RP: reversed phase
SFC: supercritical fluid chromatography
*sn*
: stereospecific numbering
TG: triacylglycerol
TIMS: trapped ion mobility spectrometry
UVPD: ultraviolet photodissociation

## 1 INTRODUCTION

**Figure 1:**
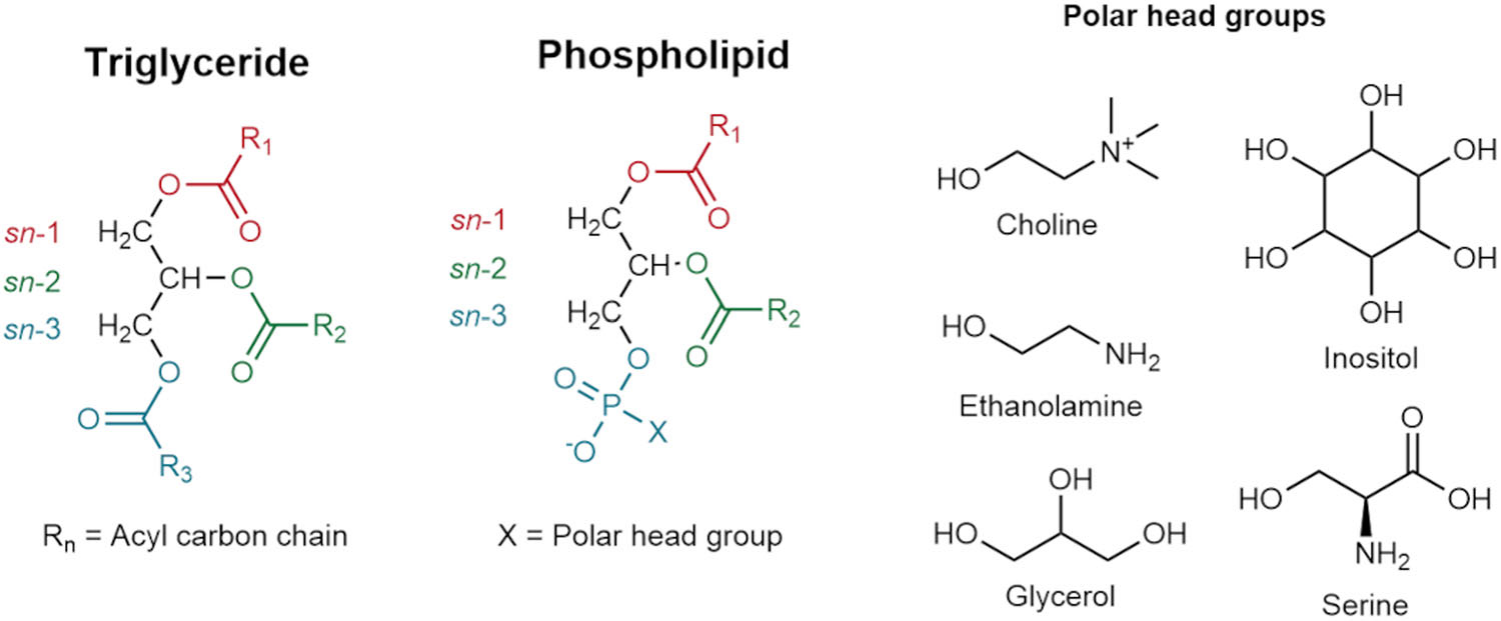
Structures of triacylglycerols and phospholipids. [Color figure can be viewed at wileyonlinelibrary.com]

Triacylglycerols (TG) are the prime constituents of natural fats and oils, while phospholipids (PL) are structural components of cell membranes. TGs consist of a glycerol backbone with three fatty acids (FA) esterified to positions *sn*‐1, *sn*‐2, and *sn*‐3 (Figure 1). The chirality of *sn*‐2 carbon in TG and PL molecules leads to formation of enantiomers. For AAB‐type TGs, where A and B represent two different FAs, there are three possible *sn*‐positional isomers, including two enantiomers. For ABC‐type TGs, there are six *sn*‐positional isomers, comprising of three enantiomeric pairs. PL structure is similar to that of TGs; however, instead of three FA, PLs consist of two FAs and a phosphate head group. If the phosphate group is assumed to be in *sn*‐3 position, there are two possible *sn*‐positional isomers for PLs containing two different FAs.

While there is evidence on the health effects of individual FAs, the effect of their stereospecific location in the glycerol backbone has been studied to a lesser extent. With dietary TGs it has been shown that FAs located in *sn*‐2 position are more readily absorbed, influencing nutritional properties particularly in infants and young animals (Bar‐Yoseph et al., 2013; Lasekan et al., 2017; Linderborg & Kallio, 2005; López‐López et al., 2001). In infant nutrition, especially the positional distribution of palmitic acid affects lipid metabolism and has implications for absorption and digestion (Innis, 2011). Long‐term feeding of TGs with different structures containing polyunsaturated FAs has been shown to impact plasma lipid concentrations in animals (Ikeda et al., 1998). Lipids with a specific structure, featuring medium‐chain FAs in the *sn*‐1 and *sn*‐3 positions and essential FA in the sn‐2 position, played an important role in providing nutritional assistance to animals suffering from malabsorption issues (Straarup & Høy, 2000). In addition, TG regioisomer structure influences fat crystallization, affecting texture and melting behavior of fats (Minato et al., 1997; Watanabe et al., 2018), and adjusting the molecular structure of fat ingredients can have a noticeable effect food production and storage (Watanabe et al., 2021). Additionally, FA chain length differences and regioisomerism impact polymorphism and oxidative stability of fat (Bouzidi & Narine, 2012). Highly polyunsaturated FAs like docosahexaenoic acid are more stable in the *sn*‐2 position (Damerau et al., 2023; Wijesundera et al., 2008), and similar results have been reported for other unsaturated FAs (Dote et al., 2016).

In case of dietary PLs, the health effects have mostly been studied on class level, and the role of individual PL molecular species remain largely to be investigated (Küllenberg et al., 2012; Skórkowska‐Telichowska et al., 2016; Sun et al., 2018; Wang et al., 2018). More interestingly, PLs have a multitude of important biological functions, and they also have potential to be used as diagnostic biomarkers of health and diseases. PLs play crucial roles in various biological processes, such as providing membranes for protein synthesis and export, maintaining cholesterol balance, and managing TG storage and secretion (Lagace & Ridgway, 2013). Disruptions in PL metabolism are associated with lipid storage issues and stress responses, ultimately contributing to health conditions such as obesity, diabetes, atherosclerosis, and neurological disorders (Dai et al., 2021; Lagace & Ridgway, 2013). Lipidomics is a rapidly expanding field of research focused on the comprehensive analysis of lipids to better understand their functions and health impacts. Despite this growth, the biological significance of individual phospholipid compounds, particularly the effects of PL regioisomers, remains largely unexplored due to a lack of powerful analytical methods to determine the regioisomeric composition of natural lipids. Recently, human breast cancer and lung cancer cells were found to contain certain phosphatidylcholine regioisomers at different ratios compared to adjacent healthy tissue (Cao et al., 2020). Comprehensive regio‐ and stereospecific structural characterization of TGs and PLs in natural samples is a challenging task primarily because there are many possible FA combinations, which can result in hundreds of individual regioisomers. Qualitative determination of molecular species of TGs or PLs is reasonably straightforward with tandem mass spectrometric methodologies. Going deeper into the structural analysis of TGs by assigning the *sn*‐positions of each FA and accurately determining ratios of the isomers is a much more demanding task.

Generally, analysis of TG and PL *sn*‐positional isomers can be divided into two main categories: the first being direct separation either by chromatographic or ion mobility techniques. Untargeted separation of isomers in natural samples is difficult, and most existing methods can only achieve partial or specific separation of individual compounds of interest. The second category is identification utilizing differential fragmentation efficiency of FAs from different *sn*‐positions. TG and PL regioisomers can be identified with MS^*n*^ methods, but analysis of TG enantiomers is not possible because the fragmentation methods cannot distinguish between *sn*‐1 and *sn*‐3 FAs due to the identical fragmentation efficiencies of FAs from these positions. The intensities of the resulting fragment ions reflect the FA distribution in the glycerol backbone. Many isobaric TG species, including isomers, are often chromatographically overlapping and may produce shared isobaric fragment ions, making the analysis of TG regioisomers even more challenging. For PLs the situation is somewhat less complicated as they can only contain up to two different FAs, resulting in significantly less interfering isobaric fragments. However, adding to the complexity of analysis of PLs is the different head groups of each class, influencing the fragmentation behavior. Traditional separation methods relying on column chromatography alone for analysis of regioisomers are becoming increasingly less common, especially when trying to achieve comprehensive identification of a wide range of regioisomers; instead, many current applications are shifting towards MS^*n*^ fragmentation and identification. Separation by ion mobility is also an emerging technique for analysis of lipid isomers.

Analysis of TG and PL *sn*‐positional isomers is not covered by standard lipidomics approaches as has been reviewed recently (Heiles, 2021; Züllig & Köfeler, 2021), highlighting the need for further development and integration of untargeted regioisomer analysis methods for lipidomics protocols. This review focuses on the liquid chromatographic, mass spectrometric, and liquid chromatographic‐mass spectrometric methodologies for analysis of *sn*‐positional isomers, including regioisomers and enantiomers of TGs and PLs as the dominant groups of lipids in natural fats and oils and cell membranes. The review covers the chromatographic separation, ionization, fragmentation, and identification as well as the challenges related to these key aspects of lipid analysis. Regioisomeric analysis of diacylglycerols and lysophospholipids, as well as determination of isomers differing in the position and configuration of double bonds are not covered in this review.

## 2 LIPID NOMENCLATURE

Shorthand notations of FA, TG, and PL follow the updated LIPID MAPS classification (Liebisch et al., 2020). Briefly, TGs are annotated based on the level of structural information as follows: species level (total number of acyl carbons and double bonds [ACN:DB] is known, e.g., TG 52:2), molecular species level (FA composition is known, but structural location is unknown, TG 16:0_18:1_18:1), regioisomer level (FA composition and *sn*‐2 FA are known, TG 16:0_18:1(*sn‐*2)*_*18:1) and enantiomer level (structural location of all three FAs is known, TG 16:0/18:1/18:1). Similarly, PLs are annotated at species level (PC 34:1), molecular species level (PC 16:0_18:1) and regioisomer level (PC 16:0/18:1). Many referenced figures from other publications use different terminology for shorthand naming. For example, AAB/ABA refers to regioisomers, where A and B are different FAs, only providing location of the *sn*‐2 FA, whereas the *sn*‐1 and *sn*‐3 FAs cannot be distinguished from one another. *sn*‐AAB/*sn*‐BAA/*sn*‐ABA means that the *sn*‐position of each FA is known.

## 3 TG

### 3.1 Separation of TG *sn*‐positional isomers

#### 3.1.1 Reversed‐phase (RP) chromatography

RP chromatography, especially with C18 columns, is the most commonly used method for separating TGs in natural samples. The C18 column, or octadecylsilane, consists of alkyl chains of 18 carbon atoms bound to silica. The octadecyl chains are hydrophobic, making the stationary phase a common choice for analysis of lipids with hydrophobic FAs. The separation is primarily governed by the equivalent carbon number (ECN) of the TG, which is defined as ACN – (2 × DB). TGs with the same ECN typically elute in clusters, and many are at least partially overlapping with each other (Ovčačíková et al., 2016).

Comprehensive separation of TG regioisomers in sources of natural origin is difficult to achieve with C18 columns, and the role of this technique combined with MS methodologies has recently been mainly for TG species profiling and qualitative identification of molecular species based on the fragment ion spectra (Li et al., 2020; Zhu et al., 2021). Specific applications for separation of TG regioisomers have been developed over the years using C18 columns, including analysis of various TG reference standards with four different columns (Momchilova et al., 2006), vaccenic and oleic acid‐containing TGs (Leskinen, Suomela, Yang, et al., 2010) and hexadecenoic acid containing TGs (Řezanka et al., 2022). Oxidized forms of TG regioisomers have also been analyzed by coupling two Kinetex C18 columns (100 × 2.1 mm, 1.7 µm) in a series (Suomela et al., 2011). The resolution of TG regioisomer separation with C18 columns is strongly influenced by the nature of the attached FAs. Generally, TG regioisomers containing FAs with higher degree of unsaturation and longer acyl carbon chains are easier to resolve from one another, whereas TGs containing only saturated or monounsaturated FAs are more difficult to separate. It was observed that the regioisomer with an unsaturated FA in *sn*‐2 and two palmitic acids in *sn*‐1/3 positions eluted earlier than the *sn*‐1/3 unsaturated regioisomer. The choice of column and the stationary phase also had a noticeable effect, and presence of higher number of residual silanol groups in the supporting material was attributed to increased TG regioisomer resolution (Momchilova et al., 2006).

**Figure 2:**
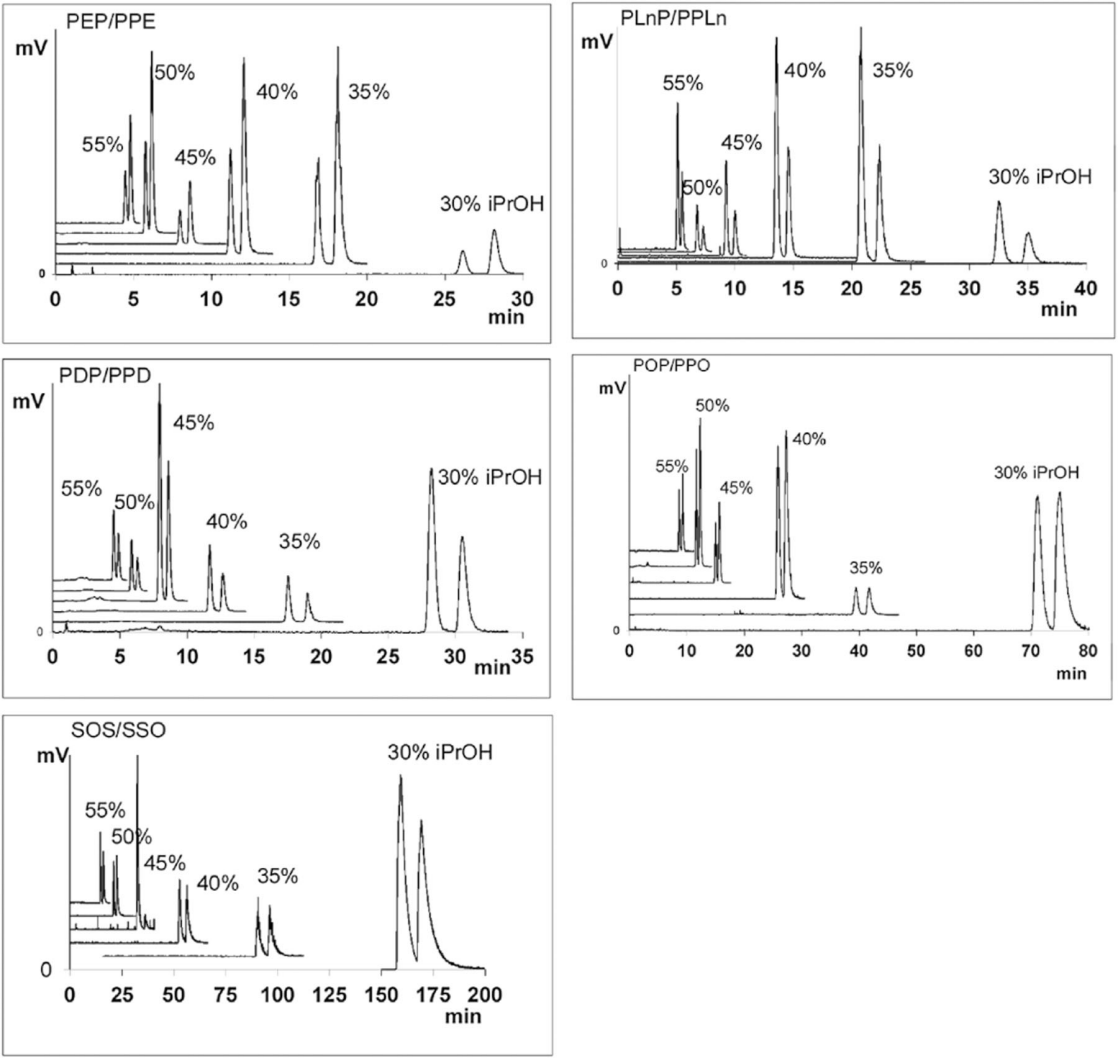
Effect of percentage of isopropanol in acetonitrile/isopropanol mobile phase on separation of regioisomers TG 16:0_20:5(*sn‐*2)_16:0/TG 16:0_16:0(*sn*‐2)_20:5 (PEP/PPE), TG 16:0_22:6(*sn‐*2)_16:0/TG 16:0_16:0(*sn‐*2)_22:6 (PDP/PPD), TG 16:0_18:3 (*sn‐*2)_16:0/TG 16:0_16:0 (*sn‐*2)_18:3 (PLnP/PPLn), TG 16:0_18:1(*sn‐*2)_16:0/TG 16:0_16:0(*sn‐*2)_18:1 (POP/PPO), TG 18:0_18:1(*sn‐*2)_18:0/TG 18:0_18:0(*sn‐*2)_18:1 (SOS/SSO) separations. Nucleodur C18 ISIS (50 × 2 mm, 1.8 µm). Reprinted with permission from Tamba Sompila et al. (2017), copyright 2017 Elsevier.

Polymeric columns, such as Nucleodur C18 ISIS with a C18/C11‐OH mixed acyl stationary phase has been particularly effective for TG regioisomer separation in some cases compared to monomeric C18 columns (Figure 2). Using isocratic elution with varying ratios of acetonitrile/isopropanol mobile phase, a near baseline separation for various TG regioisomer pairs was achieved usually in less than 10 min elution with a higher 55% isopropanol concentration. Reducing the amount of isopropanol in the mobile phase further enhanced separation, but also increased retention time. The authors noted that the presence of hydroxyl groups in the stationary phase was important for the high separation efficiency and steric selectivity when analyzing TG regioisomers (Tamba Sompila et al., 2017). Two Nucleodur C18 ISIS (250 × 2.1 mm, 1.8 μm) columns were used for a more comprehensive lipid class analysis, including some TG regioisomer pairs (Causevic et al., 2021).

C28 columns have also been used for TG regioisomer separation, mainly for pairs containing two palmitic acids and one unsaturated FA. Lowering the temperature of the column to 10–15°C was critical to achieve good chromatographic resolution for the studied TG standards. Interestingly, it was noted that separation of a regioisomer pair with two unsaturated FAs, such as TG 18:1_18:1(*sn‐*2)_16:0 and TG 18:1_16:0(*sn‐*2)_18:1 was not achieved even though they could be separated with a C18 column (Nagai et al., 2015; Nagai, Gotoh, et al., 2011). While it has some specific applications, RP chromatography alone is generally not powerful enough for separation of TG regioisomers in complex samples, but it is a very useful tool for separation of TG species for further MS‐based detection and identification.

#### 3.1.2 Silver ion chromatography

Silver ion liquid chromatography (Ag‐HPLC) in lipid analytics relies on the interactions of silver ions immobilized in the column stationary phase with the double bonds of the FAs. The higher number of double bonds means stronger interactions of the analyte with the stationary phase. Silver ions can be introduced to a column for example with silver nitrate solution, in which case the separation is also governed by the respective stationary phase of the column, for example, RP C18 (Dobson et al., 1995). However, addition of silver ions to the mobile phase is not compatible with mass spectrometry due to ion suppression and contamination of the ion source with nonvolatile compounds (Holčapek & Lísa, 2017). Instead, cation exchange columns in the stationary phase are currently the column type of choice for TG regioisomer analytics when using Ag‐HPLC (Chen et al., 2020; Lísa et al., 2011; Santoro et al., 2018). No silver ions are needed in the mobile phase with cation exchange columns as they are strongly embedded in the stationary phase, resulting in minimal leakage, and making the application suitable for MS detection (Holčapek & Lísa, 2017). Self‐modified cation exchange columns embedded with silver ions have been used for TG regioisomer separation (Leskinen et al., 2008, 2009; Santoro et al., 2018). ChromSpher Lipids (250 × 4.6 mm, 5 μm) has been a popular Ag‐HPLC column choice for TG regioisomer separations (Chen et al., 2020; Lísa et al., 2013), sometimes two (Adlof & List, 2004) or even three (Holčapek et al., 2010; Lísa et al., 2009, 2011) of them coupled together in a series.

**Figure 3:**
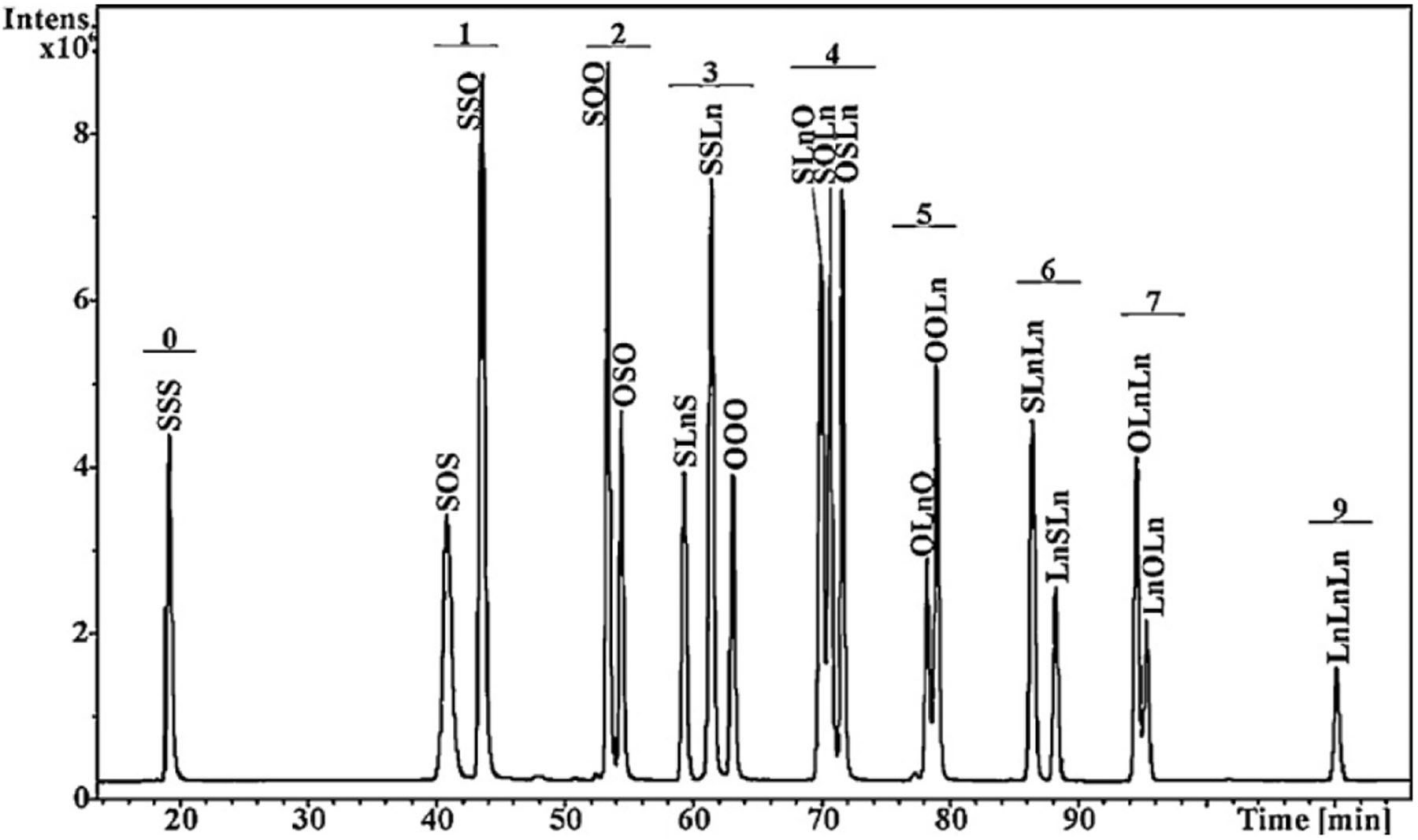
Silver‐ion HPLC/APCI‐MS chromatogram of the randomization mixture prepared from tristearin (TG 18:0/18:0/18:0), triolein (TG 18:1/18:1/18:1), and trilinolenin (TG 18:3/18:3/18:3) using three ChromSpher Lipids (250 mm × 4.6 mm, 5 µm) columns. Numbers correspond to the double bond number. Reprinted with permission from Holčapek et al. (2010), copyright 2010 Elsevier. APCI‐MS, atmospheric pressure chemical ionization‐mass spectrometry; HPLC, high‐performance liquid chromatography.

The effect of column temperature on retention times has been investigated using two ChromSpher Lipids (250 × 4.6 mm, 5 μm) columns, resulting in an interesting observation that higher temperatures significantly increased retention time for more unsaturated compounds, also increasing chromatographic resolution of TG regioisomers, for example, regioisomers of molecular species TG 18:0_18:0_18:3 and TG 18:1_18:2_18:2 (Adlof & List, 2004). This effect is opposite to the usual temperature effect in LC, which typically results in faster retention times at elevated temperatures. The increased temperature retention effect was less pronounced for TGs with lower degree of unsaturation, such as TG 16:0_16:0_18:1. It was noted that this effect is likely limited to hexane‐based solvent systems. The authors suggested that the inverse retention behavior may have been caused by a temperature‐induced change in the acetonitrile/Ag^+^ complex, which is presumably exothermic. The acetonitrile/Ag^+^ complex might be less stable in higher temperatures, allowing more interactions between the complex and the double bonds of the FAs, resulting in increased retention (Adlof & List, 2004). Similar effect was observed in another study, where retention times of TGs increased with higher temperature in the hexane mobile phase, while an opposite effect was observed for the dichloromethane mobile phase (Lísa & Denev, & Holčapek, 2013). Separation of various TG regioisomers obtained by randomization of TG 18:0/18:0/18:0, TG 18:1/18:1/18:1, and TG 18:3/18:3/18:3 is presented in Figure 3. Adequate separation of all regioisomers was achieved using three ChromSpher Lipids (250 mm × 4.6 mm, 5 µm) columns (Holčapek et al., 2010). Clear separation into groups differing in the number of double bonds was observed in all mixtures of randomized TGs.

Even with the more modern Ag‐HPLC columns and methodologies, comprehensive separation of a wide range of TG regioisomers remains challenging and time‐consuming, and the role of this separation technique has remained as a more targeted method for specific regioisomers of interest (Chen et al., 2020; Santoro et al., 2018), as molecular species might need very different chromatographic conditions for adequate regioisomer separation based on their FA compositions and degree of unsaturation. One of the main challenges of Ag‐HPLC is also poor reproducibility using conventional hexane mobile phases. Adding isopropanol to the mobile phase increased reproducibility by improving miscibility of the commonly used hexane and acetonitrile (Holčapek et al., 2010). Additionally, the fact that the separation is governed by the number of double bonds of the FAs makes separation of TGs containing only saturated FAs unviable. Ag‐HPLC can offer complementary separation capabilities when used together with RP chromatography in two‐dimensional applications. RP separates TGs mainly according to the ECN, while retention with Ag‐HPLC is mainly influenced by the number and geometry of DBs (Holčapek et al., 2009)

#### 3.1.3 Chiral phase chromatography

Chiral chromatography is a powerful technique for separating *sn*‐positional isomers of TGs. By employing a chiral stationary phase, this methodology allows the separation of TG isomers based on the spatial arrangements of the attached FA chains. Generally, the chiral recognition is based on the formation of diastereomeric complexes with different stability between the chiral selector in the stationary phase and the separated enantiomers (Alvarez‐Rivera et al., 2020). There are various chiral stationary phases utilizing different chiral selectors, resulting in complex retention mechanisms, which have been reviewed previously (Cavazzini et al., 2011). For TGs, chiral phase chromatography is currently the only chromatographic technique capable of separating enantiomers while also being capable of separating regioisomers. Current MS‐based fragmentation methods cannot differentiate enantiomers due to identical fragmentation efficiencies of *sn*‐1 and *sn*‐3 FAs, making chiral phase chromatography an important technique for this type of work. Very long analysis times are typical for chiral separations of TG isomers, and sometimes a sample recycling system is used, where the sample is repeatedly cycled through the column (Nagai, Mizobe, et al., 2011) or two columns (Kalpio et al., 2015, 2021; Nagai et al., 2015) to obtain acceptable peak resolution.

The performance of different chiral columns (CHIRALPAK IA, IB, IC, AD‐H, AS‐H, and AY‐H; CHIRALCEL OD‐H, OJ‐H, OZ‐H AD‐RH, AS‐RH, OD‐RH, and OJ‐RH) using methanol or acetonitrile as the mobile phase has been evaluated for separation of TG enantiomers. Only the CHIRALCEL OD‐RH (150 × 4.6 mm, 5 µm) with methanol as the mobile phase slightly separated the TG 16:0/16:0/18:1 and TG 18:1/16:0/16:0 from one another. Multiple passes in a recycling system were required for an adequate resolution between the two peaks, taking more than 2 h. It seems that not only is the chiral selector important for the separation, but also how it is incorporated in the stationary phase. For example, CHIRALPAK IB and CHIRALCEL OD‐RH have the same chiral selector (cellulose tris‐(3,5‐dimethylphenylcarbamate)), but the former has it immobilized in silica gel and the latter has it coated on silica gel. Adequate separation was not achieved with CHIRALPAK IB (Nagai et al., 2011).

**Figure 4:**
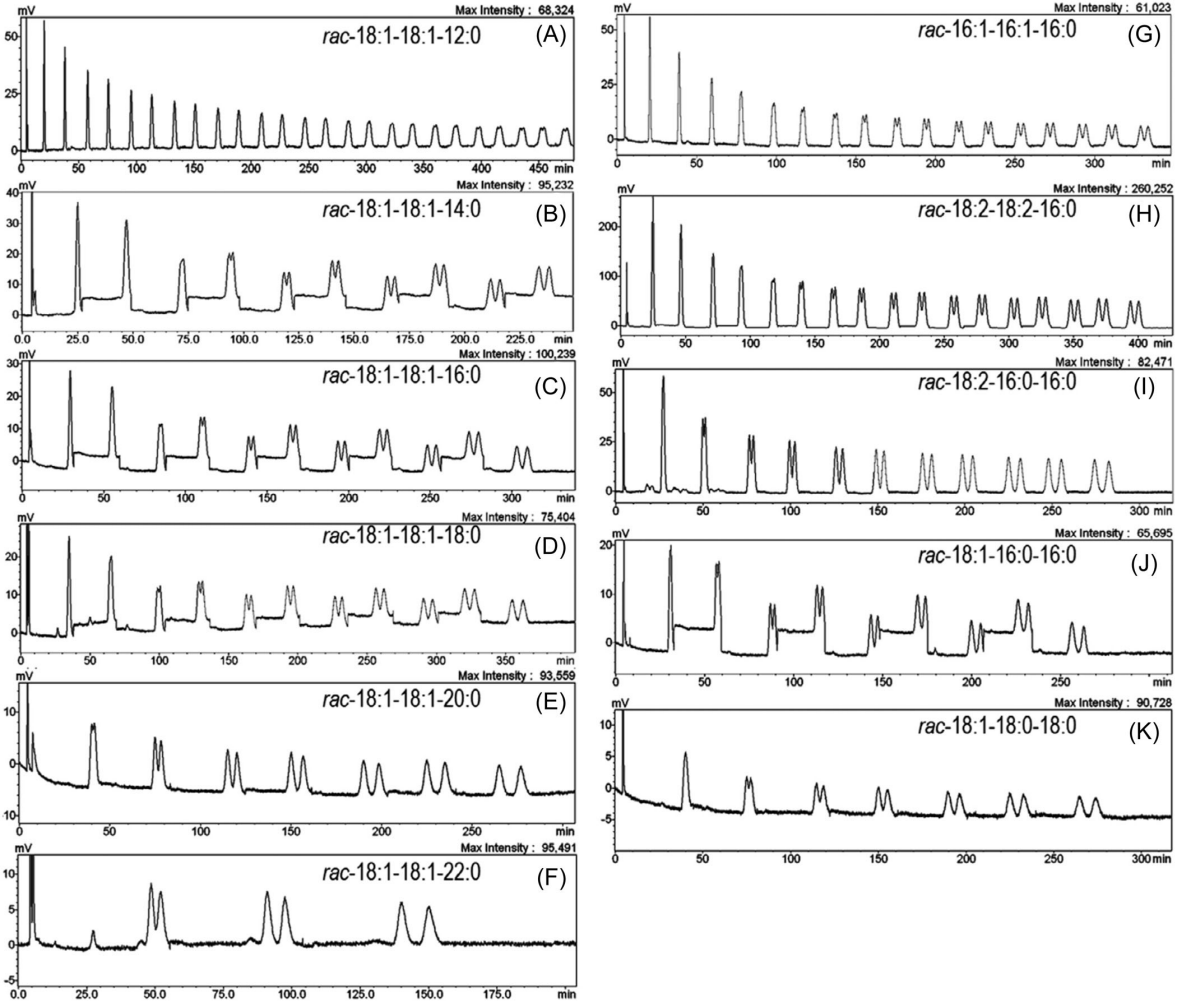
UV chromatograms of 11 racemic TG standards: TG 12:0_18:1(*sn‐*2)_18:1 (A), TG 14:0_18:1(*sn‐*2)_18:1 (B), TG 16:0_18:1(*sn‐*2)_18:1 (C), TG 18:0_18:1(*sn‐*2)_18:1 (D), TG 20:0_18:1(*sn‐*2)_18:1 (E), TG 22:0_18:1(*sn‐*2)_18:1 (F), TG 16:0_16:1(*sn‐*2)_16:1 (G), TG 16:0_18:2(*sn‐*2)_18:2 (H), TG 16:0_16:0(*sn‐*2)_18:2 (I), TG 16:0_16:0(*sn‐*2)_18:1 (J), and TG 18:0_18:0(*sn‐*2)_18:1 (K) using 2× CHIRALCEL OD‐RH (150 × 4.6 mm, 5 μm) with sample recycling and methanol as the mobile phase. Reprinted with permission from Kalpio et al. (2015), copyright 2015 Elsevier. TG, triacylglycerol; UV, ultraviolet.

The CHIRALCEL OD‐RH (150 × 4.6 mm, 5 µm) has also been used for separation and studying the retention behavior of various pure racemic TG standards found in common fats and oils (Kalpio et al., 2015). TG enantiomers were analyzed by cycling the sample through two columns until desired chromatographic resolution was achieved or it was deemed that no separation would be achieved. Eleven of the 15 racemic TGs were successfully separated. Separation of a series of racemic TGs containing dioleoylglycerol backbone and one saturated FA in *sn*‐1/3 position (TG 18:1_18:1(*sn‐*2)_X, where *X* = 12:0, 14:0, 16:0, 18:0, 20:0, or 22:0) was evaluated (Figure 4). Retention time increased and resolution of the TG enantiomers improved as the acyl chain length of the saturated FA increased. Interestingly, no enantiomer separation was achieved for TG 18:1_18:1(*sn‐*2)_18:2 containing only unsaturated FAs, whereas TG 18:0_18:1(*sn‐*2)_18:1 was successfully separated. No fully saturated TGs were analyzed in this study due to inadequate UV detector response (Kalpio et al., 2015). The same columns and sample recycling system were later used for characterization of TG enantiomers in sea buckthorn pulp oil, where careful preparative separation of TGs of interest was required for following enantiomer separations (Kalpio et al., 2021).

Previously it had been reported that both saturated and unsaturated FAs at the *sn*‐1 and *sn*‐3 would be required for separation, but TG enantiomers in bovine milk fat comprising of dipalmitoylglycerol backbone and a short‐ or medium‐chain saturated FA in *sn*‐1/3 position (TG 16:0_16:0_X, where *X* = 4:0, 6:0, 8:0, 10:0, or 12:0) have been separated using a CHIRALCEL OD‐3R (150 × 4.6 mm, 3 μm) column and a sample recycling system (Nagai et al., 2015). The CHIRALCEL OD‐3R has the same cellulose tris‐(3,5‐dimethylphenylcarbamate) coated on silica gel as the CHIRALCEL OD‐RH, with the only difference being particle size. Sufficient separation for all enantiomers of interest was achieved in five passes through the column. It was noted that the probable reason for the chiral separation was because of the CH‐π interaction between the saturated FA acyl chain and the chiral stationary phase (Nagai et al., 2015).

The enantiomeric composition of TGs consisting of C16 and C18 FAs in hazelnut oil and human plasma was analyzed with two Lux Cellulose‐1 (250 × 4.6 mm, 3 μm) columns connected in series. This was one of the first routine chiral analysis methods for TG enantiomers in natural samples. Most TGs eluted between 80 and 120 min, and while the ratios of certain enantiomers were quantified, there were several cases of co‐elution of regioisomers or enantiomers, preventing unambiguous determination of some isomer ratios (Lísa & Holčapek, 2013). Two Lux Cellulose‐1 (250 × 4.6 mm, 3 μm) in series have also been used to determine enantiomers of TG 16:0_18:1_18:2, which is the most abundant molecular species in human milk (Chen et al., 2020).

Two Astec Cyclobond I 2000 DMP (250 × 4.6 mm, 5 μm) columns in a series have been used by the same group for several studies analyzing different types of TGs of interest containing FAs such as polyunsaturated C18 FAs (Řezanka et al., 2016), C16 FAs (Řezanka & Sigler, 2014, 2022), allenic and acetylenic FAs (Palyzová & Řezanka, 2020b) and cyclofatty acids (Palyzová & Řezanka, 2020a). The chiral stationary phase of the column is 3,5‐dimethyl‐phenylcarbamate‐modified β‐cyclodextrin. The mobile phase used with the Astec Cyclobond columns in these studies was mostly hexane with a small fraction of isopropanol, usually around 1% or less at any given time during the gradient. Isopropanol was found to be important for controlling retention time, resolution, and reproducibility (Řezanka & Sigler, 2014). Enantiomers containing a more highly unsaturated FA in *sn*‐1 position was found to elute earlier (Řezanka et al., 2016). As with most other chiral chromatography applications, retention times are long, and depending on the compounds of interest, acceptable separation might take anywhere from 70 to 90 min (Palyzová & Řezanka, 2020a, 2020b; Řezanka et al., 2022) up to several hours (Řezanka & Sigler, 2014, 2016).

The performance of more recently developed CHIRALPAK ID, IE and IF columns was evaluated for TG enantiomer and regioisomer separation. CHIRALPAK IF‐3 (250 × 4.6 mm, 3 µm) column showed notably improved selectivity for TG isomers compared with the older generation CHIRALPAK OD‐RH columns, enabling acceptable resolution of various TG enantiomers and regioisomers in just one pass without sample recycling, and significantly faster (Nagai et al., 2019). For example, TG 16:0_16:0_18:1 regioisomers and enantiomers were sufficiently separated in less than 30 min with the CHIRALPAK IF‐3 column, whereas with the older CHIRALPAK OD‐RH it took more than 2 h and sample recycling to achieve similar resolution (Kalpio et al., 2015; Nagai et al., 2011).

Overall, chiral chromatography can be a powerful, albeit often time‐consuming tool for separating *sn*‐positional isomers of TGs. As MS methodologies cannot distinguish FAs dissociated from *sn*‐1/3 positions, chiral chromatography is also one of the rare applications that can be used to analyze TG enantiomers. While sample recycling through multiple columns is sometimes employed with chiral chromatography, this prevents the use of MS for monitoring the separation between the columns, as the detector needs to be nondestructive. UV detector is often employed for this purpose, while MS can be used at the end for regioisomer identification once desired separation is achieved.

#### 3.1.4 Supercritical fluid chromatography (SFC)

**Figure 5:**
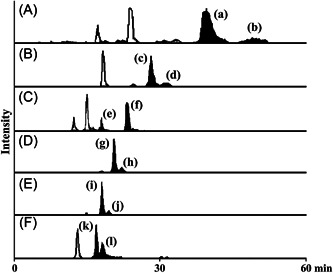
MRM chromatogram of six regioisomeric TG pairs (A–F) in palm and canola oils: (a) TG 18:0_18:1(*sn‐*2)_18:0, (b) TG 18:0_18:0(*sn‐*2)_18:1, (c) TG 18:0_18:1(*sn‐*2)_16:0, (d) TG 18:0_16:0(*sn‐*2)_18:1, (e) TG 18:0_18:3(*sn‐*2)_16:0, (f) TG 18:0_16:0(*sn‐*2)_18:3, (g) TG 16:0_18:1(*sn‐*2)_16:0, (h) TG 16:0_16:0(*sn‐*2)_18:1, (i) TG 16:0_18:2(*sn‐*2)_16:0, (j) TG 16:0_16:0(*sn‐*2)_18:2, (k) TG 16:0_18:3(*sn‐*2)_16:0, and (l) TG 16:0_16:0(*sn‐*2)_18:3 using YMC Carotenoid C30 (250 × 4.6 mm, 4 μm) column. Reprinted with permission from Lee et al. (2014), copyright 2014 Elsevier.

In recent years, SFC has seen an increase in usage in the field of lipidomics (Lísa & Holčapek, 2015; Takeda et al., 2018; Wolrab et al., 2020) and TG profiling (Tu et al., 2017; Zhang et al., 2019, 2021), including some regioisomeric separation methods (Lee et al., 2014; Masuda et al., 2021). SFC employs supercritical fluid, typically carbon dioxide, as part of the mobile phase. There are several reasons why carbon dioxide is the most widely used supercritical fluid in SFC, such as miscibility with most organic solvents used in the mobile phase and the critical values of pressure and temperature being easy to reach. Some additional benefits of carbon dioxide are affordability, nonflammability, and limited toxicity (West, 2018). Six TG regioisomer pairs found in palm and canola oils have been successfully separated using YMC Carotenoid C30 (250 × 4.6 mm, 4 μm) column (Figure 5).

Two chiral phase columns CHIRALPAK IG‐U (100 × 3 mm, 1.6 μm) and CHIRALPAK IG‐U (150 × 3 mm, 1.6 μm) in series were used to separate both enantiomers and regioisomers of TG 16:0_18:1_18:1 for the first time using SFC (Masuda et al., 2021). Suitability of different organic solvent modifiers such as methanol, ethanol, isopropanol, and acetonitrile was screened, and a mixture of 90% acetonitrile and 10% methanol was selected. Near baseline separation of enantiomers TG 16:0/18:1/18:1 and TG 18:1/18:1/16:0 was achieved in 35 min. The regioisomer TG 18:1/16:0/18:1 was partially overlapping with TG 18:1/18:1/16:0, but the resolution was sufficient for quantification purposes (Masuda et al., 2021). Recently, five RP columns in a series (four Kinetex C18 (150 × 4.6 mm, 2.6 μm) and one Accucore C18 (150 × 4.6 mm, 2.6 μm) were used for identification of TGs of a wide variety of vegetable fats and oils using SFC. While analysis of TG regioisomers was not the main objective of this study, several TG regioisomer pairs were resolved chromatographically (Gros et al., 2023). SFC for the separation of TG regioisomers remains relatively unused. Most existing applications primarily focus on high‐throughput general lipidomics with MS identification. However, the rapid advancements and growing popularity of SFC instrumentation warrant a more in‐depth exploration of its potential for separating TG regioisomers.

#### 3.1.5 Two‐dimensional chromatography

A two‐dimensional chromatography system consists of two columns, typically with orthogonal selectivity. The peaks of interest separated in the first dimension can be further directed into the second dimension, which will separate the compounds that were co‐eluting in the first dimension. Two‐dimensional chromatography has been used for analysis of TG regioisomers, commonly comprising of a silver ion and a RP column. There does not seem to be a clear preference on which technique to start with, as researchers are using silver ion followed by RP chromatography (Arena et al., 2021; Mondello et al., 2005; Yang et al., 2012) and vice versa (Chen et al., 2019; Dugo et al., 2004; Holčapek et al., 2009). Depending on the complexity of the sample and the compounds of interest, sometimes it might be more practical to start with silver ion chromatography to get first dimension separation based on the number of double bonds, or sometimes starting with separation by ECN using RP can be useful. Two‐dimensional chromatography can be done either off‐line or on‐line. Off‐line two‐dimensional chromatography means that the peaks of interest are fractionated in the first column, collected, and then reinjected to the other column in a separate analysis. On‐line two‐dimensional chromatography uses a series of valves, tubing, and careful timing to direct the compounds from the first dimension to the second within the same analysis. For two‐dimensional systems using silver ion and RP chromatography for TG regioisomer separation, some are off‐line (Chen et al., 2019; Dugo et al., 2004; Holčapek et al., 2009), and some on‐line methods (Arena et al., 2021; Mondello et al., 2005; Yang et al., 2012). There are also several examples of using a RP C18 column for preparative molecular species or regioisomer separation and fraction collection, followed by chiral phase chromatography for enantiomer separation (Chen et al., 2020; Kalpio et al., 2015, 2021; Nagai et al., 2011).

While two‐dimensional chromatography can offer enhanced separation of TG regioisomers compared to more conventional chromatographic applications, it also has some drawbacks. Off‐line two‐dimensional chromatography is cumbersome, requires precise timing for fraction collection, and is not well suited for high‐throughput applications. On‐line two‐dimensional chromatography necessitates specialized instrumentation and generally demands more expertise compared to conventional chromatography, which can limit its widespread adoption. Furthermore, solvent compatibility between the two dimensions can pose some challenges. Great care should be used when selecting the solvent compositions and flow rates. As separation of TG regioisomers is rather delicate, excessive or too strong eluent from the first dimension may negatively impact peak shape in the second dimension, potentially decreasing the performance of the overall method.

#### 3.1.6 Ion mobility

Ion mobility as a separation technique for analysis of TGs has seen an increase in relevance over the recent years, allowing rapid separation of ions moving through inert gas buffer in an electric field. Separation with ion mobility is based on differences in the collisional cross section (CCS) of the analytes. Molecules with higher CCS values encounter higher resistance as they are moving through the gas phase. Different three‐dimensional configurations of TG isomers result in slightly different CCS values, potentially enabling separation of the isomers. Current ion mobility applications capable of separating TG regioisomers are limited in numbers, but the potential exists.

To our knowledge the first study to separate TG regioisomers with ion mobility employed differential mobility spectrometry (DMS) (Šala et al., 2016). This study also investigated several factors influencing the separation, such as adduct type, chemical modifier composition, chemical modifier flow rate, separation voltage and pressure of the compensation gas. No separation was observed for the [M+Na]^+^ or [M+NH_4_]^+^ adducts of regioisomers TG 18:0_18:1(*sn‐*2)_18:1 and TG 18:1_18:0(*sn‐*2)_18:1 with the initial settings. [M+Ag]^+^ adducts, however, were possible to separate, and were selected for further optimization. Both chemical modifier composition and flow rate had significant effect on the ion mobility separation, and out of the tested methanol, ethanol, isopropanol, 1‐propanol, and 1‐butanol, certain solvents were more suitable depending on whether resolution or sensitivity was prioritized. 1‐Propanol was better for sensitivity while providing acceptable resolution, whereas 1‐butanol provided better resolution at the cost of sensitivity. Higher separation voltages increased regioisomer resolution. While higher throttle gas pressure also resulted in better separation, it also significantly reduced sensitivity (Šala et al., 2016). In another study, DMS was used to test separation of various lipid isomer standards, including regioisomers TG 16:0_18:1(*sn‐*2)_20:0 and TG 16:0_20:0(*sn‐*2)_18:1, achieving partial separation (Bowman et al., 2017).

Recently, our group had the opportunity to test TG regioisomer separation of various standards with the Waters SELECT SERIES Cyclic ion mobility spectrometer (Unpublished results). The instrument has a unique feature of the possibility to recycle the ions in the circular path of the ion mobility system for improved separation. Regioisomer pairs TG 14:0_14:0(*sn‐*2)_18:1/TG 14:0_18:1(*sn‐*2)_14:0 and TG 16:0_16:0(*sn‐*2)_18:1/TG 16:0_18:1(*sn‐*2)_16:0 were nearly baseline separated, whereas pairs TG 18:1_18:2(*sn‐*2)_18:2/TG 18:2_18:1(*sn‐*2)_18:2 and TG 18:1_18:1(*sn‐*2)_18:3/TG 18:1_18:3(*sn‐*2)_18:1 were partially separated. Analyses were performed with direct infusion. Optimization of parameters was not attempted in these tests, and only [M+NH_4_]^+^ adducts were investigated (Unpublished results). An earlier study (Šala et al., 2016) suggests that further improvements to TG regioisomer separation could have been possible after thorough method optimization. So far ion mobility techniques for separating TG regioisomers have only been demonstrated on a quite narrow range of regioisomer standards. Compared to direct separation of TG regioisomers, there is likely a greater potential for ion mobility to be coupled with liquid chromatography for further separation of co‐eluting isobaric TG molecular species.

### 3.2 Identification of TG regioisomers by MS methodologies

As opposed to other methods purely relying on separation of TG isomers, MS methodologies typically utilize the differential fragmentation of FAs from *sn*‐1/3 or *sn*‐2 positions to determine the regioisomer ratios. The MS methodologies still often use chromatographic separation of TGs to reduce the number of co‐eluting TG species that might convolute the MS^*n*^ identification due to isobaric fragments from different molecular species. Some applications use direct inlet techniques without separation. Electrospray ionization (ESI) in positive polarity mode with collision‐induced dissociation (CID) is currently the most commonly used MS^2^ technique for analysis of TG regioisomers. Atmospheric pressure chemical ionization (APCI) or chemical ionization (CI) are also used in some methods. Most methods rely on one fragmentation step, but depending on instrument capabilities, several studies also utilize sequential fragmentation, commonly MS^3^. Because APCI produces abundant structurally informative in‐source fragments, it has been used for TG regioisomer analysis without a dedicated fragmentation step. Analysis of TG regioisomer ratios using MS^*n*^ methodologies typically rely on calibration curves created with regiopure reference standards. Different TG species produce fragment ions at different abundance ratios depending on their FA composition, and if these differences are not accounted for, the results may be inaccurate.

#### 3.2.1 CID

##### Mechanistic studies on fragmentation of TG regioisomers

**Figure 6:**
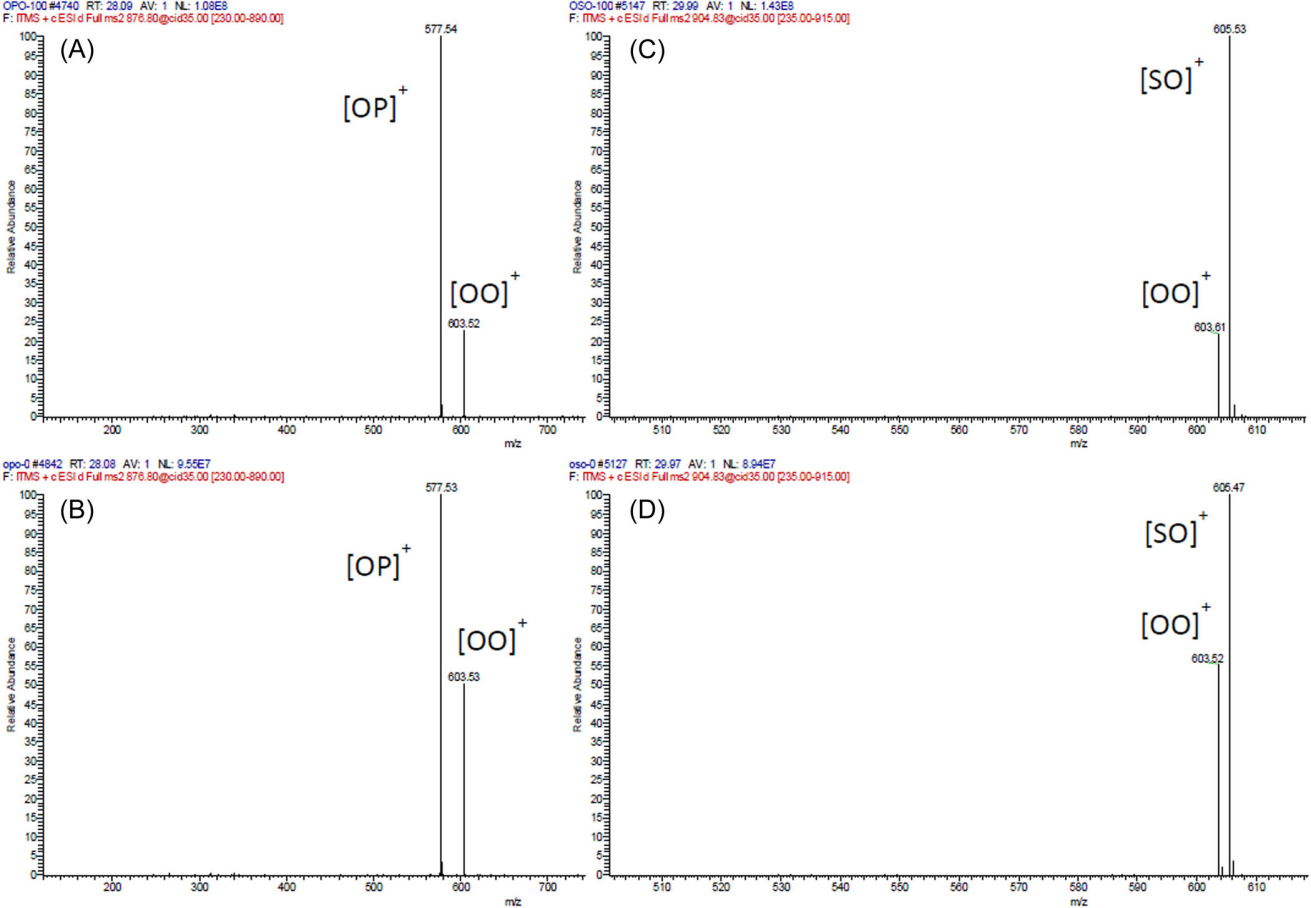
Fragment ion spectra of two TG regioisomer pairs: TG 18:1_16:0(*sn‐*2)_18:1 (A), TG 16:0_18:1(*sn‐*2)_18:1 (B), TG 18:1_18:0(*sn‐*2)_18:1 (C), and TG 18:0_18:1(*sn‐*2)_18:1 (D). Reprinted with permission from Liu and Rochfort (2021), copyright 2021 MDPI. FA, fatty acid; TG, triacylglycerol. [Color figure can be viewed at wileyonlinelibrary.com]

Different TG adducts may have distinct fragmentation patterns, some of them being better suited for TG regioisomer analysis than others. For example, using ESI–CID–MS^*n*^, different methods have used [M+NH_4_]^+^, [M+Li]^+^, [M+Na]^+^, [M+Ag]^+^, or [M+Ag+AgNO^3^]^+^ ions to produce structurally informative fragment ions. Examples of typical CID fragment spectra of two TG regioisomer pairs is displayed in Figure 6, showing the structurally relevant DG fragment ions and their relative differences in abundance ratios that can be used to establish calibration curves for regioisomer analysis. Ammoniated [M+NH_4_]^+^ precursor ions is the most commonly used adduct type for TG regioisomer analysis, and there are several studies that have investigated the CID fragmentation patterns of [M+NH_4_]^+^ ions. A series of three studies focused on oleic acid (Li & Evans, 2005), palmitic acid (Li et al., 2006), and linoleic or arachidonic acid (Gakwaya et al., 2007) containing TGs. The first study, consisting of the series with TG 18:1/X/18:1 and TG X/18:1/X (*X* = saturated or monounsaturated FAs with varying chain lengths), discovered that there were linear correlations between the fractional intensities of the various DG fragments and the chain length of the X FA (Li & Evans, 2005), indicating that fragmentation efficiencies of various TG molecular species could be predicted based on the FA composition of the TG. In the second part of the study, a series of TG 16:0/X/16:0 and TG X/16:0/X produced similar results in terms of the chain length affecting the fractional abundance of DG‐like fragments [M+NH_4_−RCO_2_H−NH_3_]^+^ with saturated or monounsaturated FAs accompanying the palmitic acid (Li et al., 2006). However, with the more unsaturated linoleate (TG 18:2/X/18:2 and TG X/18:2/X) and the arachidonate series (TG 20:4/X/20:4 and TG X/20:4/X) the effects of the chain length were less clear (Gakwaya et al., 2007). Predicting fragmentation patterns of TGs is useful, because the amount of naturally occurring TGs is vast, and the number of commercially available regiopure TG standards is relatively low.

**Figure 7:**
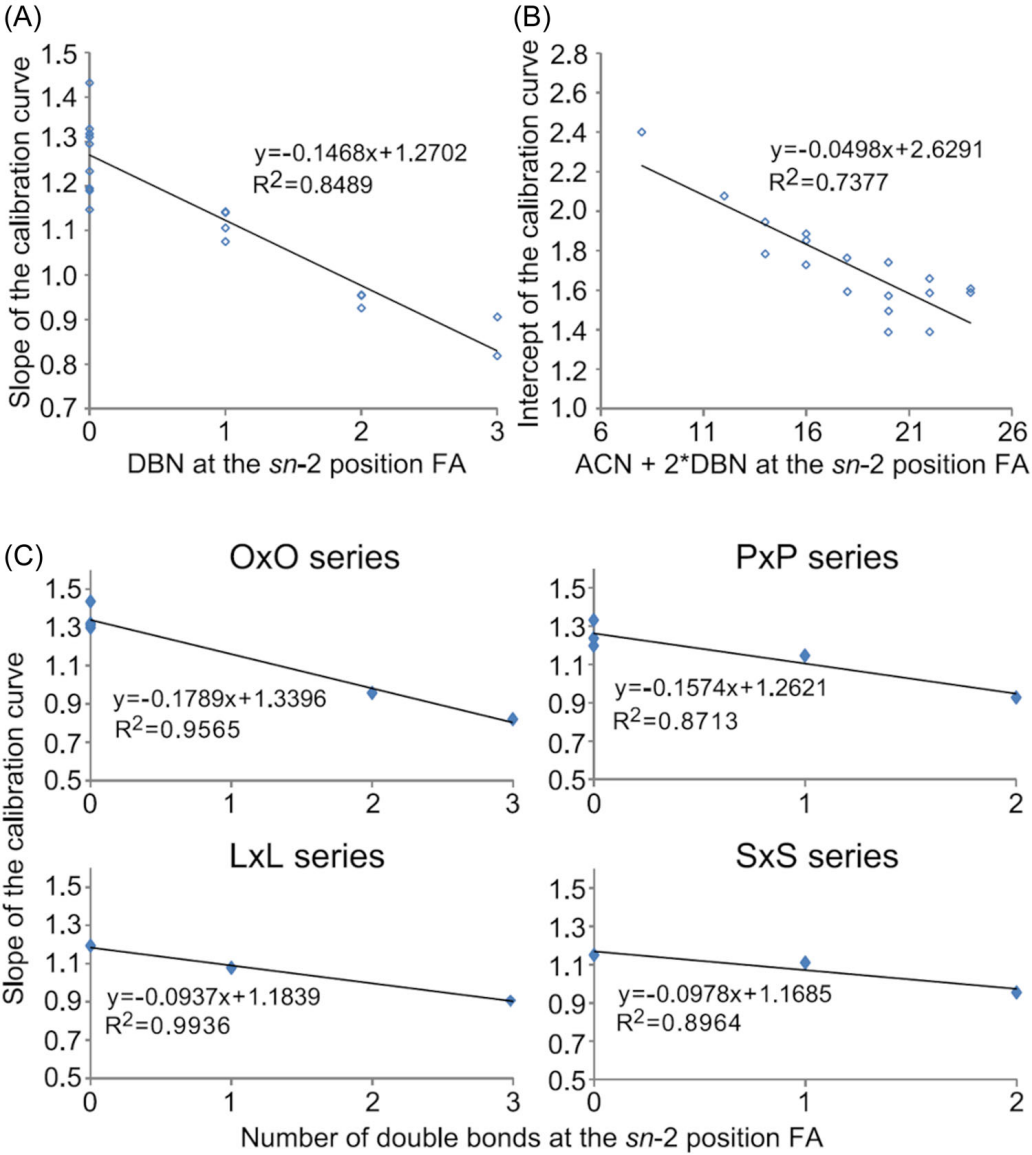
Negative linear correlation between the DB in the *sn*‐2 FA and the slope of the calibration curve (A). Negative linear correlation between the ACN + 2 × DB and the intercept of the calibration curve (B). Negative linear correlations between the DB at the *sn*‐2 FA and the slope of the calibration curve when the TGs are grouped in four groups having the same primary position FAs within groups (C). Reprinted with permission from Tarvainen et al. (2019), copyright 2019 American Chemical Society. [Color figure can be viewed at wileyonlinelibrary.com]

The fragmentation efficiencies of [M+NH_4_]^+^ adducts of various TGs were also studied after a synthesis of a wide range of regioisomer reference standards. Presence of multiple unsaturated FAs decreased the slopes of the calibration lines closer to zero, especially for TGs containing two arachidonic or docosahexaenoic acids, meaning that the fragmentation is less regiospecific. This caused uncertainties in determining the regioisomer ratios, as the fractional abundances of the structurally informative DG fragments are relatively similar with both regioisomers (Judge et al., 2017). Negative linear correlations have been observed between the number of double bonds in the *sn*‐2 FA and the slope of the calibration curve (Figure 7) (Tarvainen et al., 2019). The correlation was much stronger when TGs were grouped in four groups having the same primary position FAs within groups. Negative linear correlation was also discovered between the ACN + 2 × DB and the intercept of the calibration curve. Combining these two observations it could be possible to create a fragmentation model (Tarvainen et al., 2019). A mechanistic study on the TG fragmentation pathways concluded that the loss of *sn*‐1/3 FAs have lower activation energies than *sn*‐2 FA. The loss of *sn*‐2 FA is, however, entropically more favorable, and is highly influenced by the FA in that position (Renaud et al., 2013).

The double bond positions in the FAs also seem to play a role in the fragmentation process. Double bonds positioned close to the carbonyl carbon along the fatty acid chain promote the formation of the DG fragment ion corresponding to the loss of the fatty acid. For example, synthetic standards TG 16:0_18:1(9Z)(*sn‐*2)_16:0 and TG 16:0_18:1(6Z)(*sn‐*2)_16:0 have distinctly different fragmentation patterns (Li et al., 2006). This creates some additional challenges as natural mixtures might contain various FA double‐bond isomers in the same TG molecular species. While current analysis methods utilizing CID and fragments of ammoniated [M+NH_4_]^+^ adducts cannot distinguish FA double bond isomers as they have the same *m/z* ratio, there are some examples where fragmentation with electron impact excitation of ions from organics (EIEIO) has been used for simultaneous assignment of both FA *sn*‐positions and locations of their double bonds (Baba et al., 2016).

**Figure 8:**
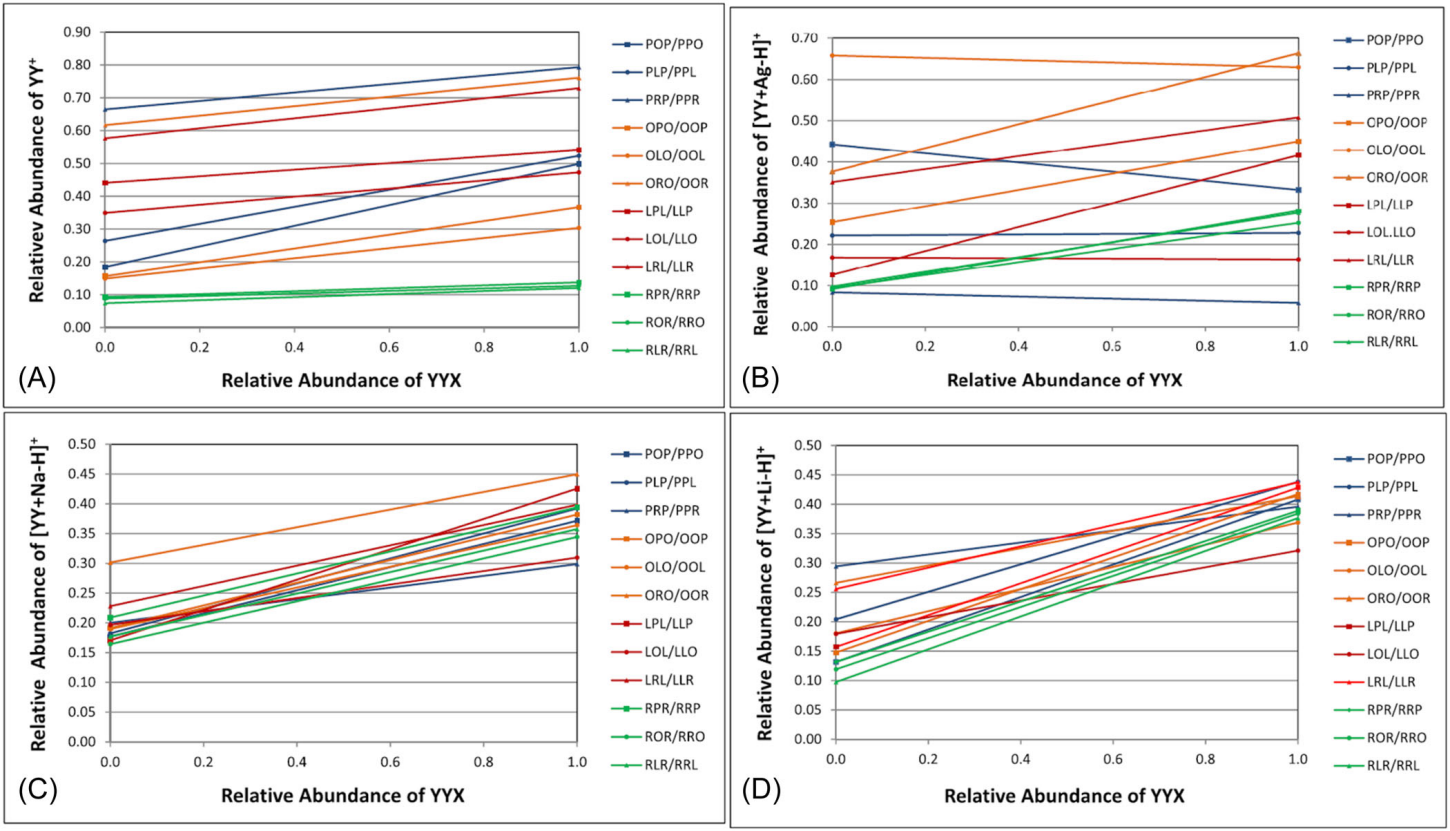
Calibration plots of relative abundances of DG‐like fragment ions for 12 TG regioisomer pairs using ammonium ion (A), silver ion (B), sodium ion (C), or lithium ion (D) as the complexing reagent. Reprinted with permission from Makarov et al. (2018), copyright 2018 John Wiley and Sons. DG, diacylglycerol; TG, triacylglycerol. [Color figure can be viewed at wileyonlinelibrary.com]

In addition to FA composition of the TG molecules, the influence of different TG adduct ion types on fragmentation efficiencies has also been studied. Positional sensitivity of CID fragments from [M+NH_4_]^+^, [M+Li]^+^, [M+Na]^+^, and [M+Ag]^+^ adduct ions was investigated in a series of two studies (Gazlay & Evans, 2022; Makarov et al., 2018). An important observation was that despite [M+NH_4_]^+^ adducts being the most frequently used in many methods and the most thoroughly studied, they might not be the most suitable ones for analysis of TG regioisomers. Fragmentation behaviors, including the relative abundances of the DG‐like fragment ions from both [M+NH_4_]^+^ [M+Ag]^+^ precursor ion adduct types were less consistent compared with fragments of [M+Na]^+^ and [M+Li]^+^ adducts (Figure 8). With [M+NH_4_]^+^ ions, some of the calibration plots had significantly higher slopes and different intercepts (Makarov et al., 2018), and as also observed in an earlier study (Gakwaya et al., 2007), the TG standards containing two arachidonic acids produced calibration plots with nearly flat slope due to low positional sensitivity of the fragmentation. The [M+Ag]^+^ adduct series was even more erratic and random compared to the [M+NH_4_]^+^ series, suggesting that an attempt to create a model for the fragmentation pattern would not be viable (Makarov et al., 2018). However, the calibration plots for [M+Li]^+^ and especially [M+Na]^+^ adducts were significantly more uniform with similar slopes and intercepts reasonably closely clustered together (Figure 8). Another earlier study investigating the same four adduct types came to the same conclusion that [M+Na]^+^ adducts are the most suitable for TG regioisomer analysis as they produced the most consistent level of positional sensitivity for the fragmentation (Ramaley et al., 2015). However, according to some previous literature, fragments of [M+Na]^+^ adducts provided limited structural information (Segall et al., 2005). This might have been caused by differences in instrumentation or collision gas used, suggesting that testing different ion complexing agents could be worth trying when developing a TG regioisomer analysis method for a specific laboratory system. While [M+Li]^+^ adducts also produce consistent calibration plots, the lithium salts used in the mobile phase are known to sometimes cause precipitation of lithium salt crystals in the instrumentation (Kallio et al., 2017).

##### Analysis of TG regioisomers using CID

Composition of human milk TGs, including tentative identifications of numerous regioisomers, has been analyzed using a nontargeted lipidomics method (George et al., 2020). While the study was able to reveal detailed TG composition and many molecular species not previously reported in human milk, the TG regioisomer identifications were simply based on identifying the least abundant DG fragment, as that is most likely the one that has lost its *sn*‐2 FA. As the ratios of DG fragments represent mixtures of TGs instead of a single isomer, the identifications were only given for the highest abundance regioisomer, and no attempt was made at determining the regioisomer ratios (George et al., 2020). Similarly, in certain studies, sometimes only one regioisomer is identified based on the relative abundances of the fragment ions, and the fact that natural TGs are commonly mixtures of regioisomers is omitted (Nguyen et al., 2018; Tu et al., 2017). While CID strongly favors dissociation of FAs from *sn*‐1/3 positions, only examining the least abundant DG fragment, which presumably has lost *sn*‐2 FA, will at best provide information on the most abundant TG regioisomer, but should not be used as the basis for excluding the presence of other isomers as seen in some previously published literature. Establishing calibration curves with regiopure reference standards for the TG regioisomers of interest is common using the fractional abundances of the DG fragment ions (Leskinen et al., 2010; Liu & Rochfort, 2021; Malone & Evans, 2004). Calibration curves for ABC‐type TGs were reported for the first time in addition to numerous AAB/ABA‐type TGs (Tarvainen et al., 2019). The effects of chromatographically overlapping isobaric fragments should be considered, as the abundance ratios of structurally informative fragments can be distorted by fragments of other co‐eluting TGs. Either acceptable chromatographic resolution between the isobaric TG molecular species (Liu & Rochfort, 2021) must be achieved, or an alternative method, such as algorithmic interpretation of the spectra (Kurvinen et al., 2001; Sazzad et al., 2022), should be employed to mitigate the interference of the overlapping fragments. In reality, algorithmic interpretation is necessary for nontargeted isomeric analysis of natural lipids, since complete separation of isobaric TG molecular species is practically not yet achievable with current methodologies. Recently, our laboratory developed an algorithmic optimization tool called *TAG Analyzer*, which utilizes a fragmentation model derived from an extensive set of standard curves. This innovative tool has demonstrated its effectiveness in determining the detailed regioisomer profile of highly complex natural fats and oils, including human milk (Sazzad et al., 2022).

Regioisomers of TGs containing vaccenic acid (FA 18:1(11Z)) and oleic acid (FA 18:1(9Z)) have been analyzed (ESI(+)‐CID‐MS^2^, [M+NH_4_]^+^) in sea buckthorn (*Hippophaë rhamnoides* L.), and the calibration curves for regioisomers of molecular species TG 16:1(9Z)_16:1(9Z)_FA 18:1(11Z) and TG 16:1(9Z)_16:1(9Z)_FA 18:1(9Z) were similar (Leskinen et al., 2010). However, minor differences were observed due to preferential loss of FA 18:1(9Z) acid compared to FA 18:1(11Z). As the MS technique used in this study was not able to distinguish the FA isomers with different double bond locations from overlapping peaks, chromatographic resolution of the two molecular species was vital (Leskinen et al., 2010).

In their work of analyzing a wide range of TG regioisomer standards, the composition of olive oil was analyzed (ESI(+)‐CID‐MS^2^, [M+NH_4_]^+^) using the established calibration curves (Tarvainen et al., 2019). Olive oil being a relatively simple mixture of TGs, there were only few chromatographically overlapping isobaric TG molecular species with interfering fragment ions, most notably TG 18:1_18:1_18:3 and TG 18:1_18:2_18:2. The solution was to select the spectra from the leading edge of the partially resolved first peak and the tailing edge of the second peak to avoid isobaric interference of the fragment ion abundance ratios. This is not ideal solution, and the edge does not represent the entire peak, as there is usually at least some chromatographic separation of the TG regioisomers in addition to the molecular species. Regioisomers of molecular species TG 16:0_18:0_18:1, TG 18:0_18:0_18:1, and TG 18:0_18:1_18:1 were analyzed (ESI(+)‐CID‐MS^2^, [M+NH_4_]^+^) from various sources, including vegetable oils and meat. Calibration curves were established with reference standards, and the authors also noted that analysis of 18:0_18:0_18:1 was not possible from the selected vegetable oils due to interfering DG fragments from other TG molecular species with the same molecular weight (Malone & Evans, 2004).

A RP C30 column (Acclaim C30 [250 × 3 mm, 3 μm]) has been used to mitigate the effects of overlapping isobaric molecular species to analyze (HESI(+)‐CID‐MS^2^, [M+NH_4_]^+^) specific TG regioisomers in bovine milk (Liu & Rochfort, 2021). In addition to separating TG 18:0_18:1_18:1 and 18:0_18:0_18:2, also double bond isomers of TG 18:0_18:1_18:1 were identified. While the major molecular species were separated, also other minor interfering TGs were observed. However, their contribution to the abundances were deemed minimal, and calibration curves could be used for quantifying the regioisomers of the major molecular species (Liu & Rochfort, 2021).

Sequential fragmentation with an ion trap instrument (ESI(+)‐CID‐MS^3^, [M+Li]^+^) has been applied for analysis of TG regioisomers in olive oil utilizing lithiated adducts. The DG‐like fragment ions [M+Li–RCOOH]^+^ from the first CID step were further fragmented, and calibration curves were established using the resulting α,β‐unsaturated FA fragments. The calibration curves were not linear, and it was suspected that this was mainly caused by the sequential two fragmentation steps instead of just one (Lin & Arcinas, 2008). Nonlinear calibration plots were also observed in another study using two‐step fragmentation of lithiated TG adducts (ESI(+)‐CID‐MS^3^, [M+Li]^+^) (Ramaley et al., 2013), suggesting that the nonlinearity of the TG calibration curves could be intrinsic, only being accentuated by the MS^3^ fragmentation. Most MS^2^ calibration plots in the literature are using linear fitting, but this might not be ideal as the nonlinearity, especially when using MS^3^, could be affected by acyl chain lengths and numbers of double bonds in the attached FAs (Ramaley et al., 2013). For example, when analyzing TG regioisomers in fish oils (ESI(+)‐CID‐MS^3^, [M+Li]^+^), the calibration plot for TG 16:0_16:0_22:6 and TG 16:0_16:0_20:5 were nonlinear (Cubero Herrera et al., 2013), suggesting that linearity or nonlinearity should be experimentally verified by assessing different fittings to the calibration data. Later, the applicability of MS^3^ without chromatographic separation for analysis (ESI(+)‐CID‐MS^3^, [M+Li]^+^) of complex TG mixtures such as fish oil was investigated (Ramaley et al., 2015). In MS^3^, the peaks arising from the loss of lactone were used for the calculations. As there is no chromatographic separation, the influence of ^13^C isotopes needed to be considered. For example, the M+2 isotope of TG 16:1_18:0_18:1 has the same m/z as TG 16:0_18:0_18:1, as the difference of one double bond in the molecular species results in the difference of 2 Da. Using MS^3^ removed the interference of isomeric TGs with one FA in common with the target analyte. In cases where the interfering isotopes had two FAs in common with the target analyte, corrections of the isotope clusters were more complicated, though possible (Ramaley et al., 2015).

Major TG regioisomers in *Mycobacterium smegmatis* biofilm were assessed (ESI(+)‐CID‐MS^3^, [M+Li]^+^) by the notion that the fragment ions arising from the loss of *sn*‐2 FA are less abundant than those of arising from loss of *sn‐*1/3 FAs. No attempt was made at determining the regioisomer ratios, and only the most abundant ones were reported (Purdy et al., 2013). Trihydroxy FA containing TG regioisomers in castor oil have been characterized (ESI(+)‐CID‐MS^3^, [M+Li]^+^). Despite the additional hydroxyl groups attached to the acyl chains of the FAs, the regioisomer ratios could be determined in a similar manner as with TGs containing only regular FAs (Lin & Chen, 2010). A kinetic method utilizing lithium as a transition metal ion to form complex ions involving both an analyte TG and a reference TG has been used to analyze (ESI(+)‐CID‐MS^3^, [M+Li+M_ref_]^+^) soya oil. The method utilizes competitive dissociation between the reference and the analyte TG. The ratio of the two competitive dissociation rates, defined by the product ion branching ratio was related via the kinetic method to the regioisomeric composition of the investigated TG mixture. Linear correlation was established between composition of the mixture of each TG regioisomer and the logarithm of the branching ratio for competitive fragmentation (Leveque et al., 2012).

While ESI is currently the dominant ionization technique for analysis of TG regioisomers, also APCI has seen some use together with CID for analysis of TG regioisomers. Selected regioisomers in human milk were analyzed on negative polarity with ammonia as the nebulizer gas to form deprotonated molecular ions (APCI(–)‐CID‐MS^2^, [M–H]^–^). Calculations of regioisomer ratios were based on the relative abundances of [M–H–100–FA]^–^ fragment ions (Linderborg et al., 2014). The method was first adapted from an old direct inlet negative ion chemical ionization method (CI(–)‐CID‐MS^2^, [M–H]^–^) (Kallio & Currie, 1993) to an LC‐MS system (APCI(–)‐CID‐MS^2^, [M–H]^–^) (Leskinen et al., 2010). TGs containing abundant long‐chain polyunsaturated FAs in tuna oil and algae oil have been analyzed (APCI(+)‐CID‐MS^2^, [M+H]^+^) and the major TG regioisomer within each co‐eluting molecular species was reported (Baiocchi et al., 2015).

#### 3.2.2 In‐source fragmentation

Some ionization techniques such as APCI produce abundant in‐source fragmentation of TG molecular ions. This feature can be utilized for structural analysis of TGs without a dedicated fragmentation step. However, due to the lack of precursor‐fragment ion relationship offered by tandem mass spectrometry, in‐source fragmentation is only suitable for simple TG mixtures or using chromatographic separation of the molecular species with overlapping DG fragments. A comparison of different mass spectrometers (APCI(+)‐MS, [M+H]^+^) for investigating TG fragmentation behavior with APCI was performed with a total of 84 synthesized TG standards. The main conclusion was that while there were minor differences between the instruments, the number of double bonds and their position in the acyl chain had significantly larger influence on the DG fragment ion ratios (Holčapek et al., 2010) The results were grouped according to the degree of saturation (saturated, monounsaturated, diunsaturated, and triunsaturated) of the individual FAs on the TGs, and good correlation was observed in the fragment ion ratios within the groups. The authors suggest that the data of TGs with specific unsaturation patterns could be generalized for other TGs with similar unsaturation pattern (Holčapek et al., 2010).

FA 20:4 and FA 20:5 containing TG regioisomer ratios, consisting of molecular species TG 16:0_16:0_20:4, TG 16:0_16:0_20:5, TG 16:0_20:4_20:4, and TG 16:0_20:5_20:5, in *Trachydiscus minutus* algal oil were estimated (APCI(+)‐MS, [M+H]^+^) after acceptable chromatographic separation (Řezanka et al., 2011). Some molecular species were only partially separated, but the resolution was good enough to evaluate the regioisomers based on the DG fragment abundances. No calibration curves were used, and the estimations relied on previous observations (Holčapek et al., 2010) of fragmentation behavior of various TG standards. A significant difference in DG fragment ion ratios with in‐source fragmentation (APCI(+)‐MS, [M+H]^+^) and CID (APCI(+)‐CID‐MS^2^, [M+H]^+^) has been observed for TGs containing one shorter and less unsaturated FA together with two long‐chain polyunsaturated FAs (Baiocchi et al., 2015). However, the fragment abundance ratios were consistent for TGs containing FAs with similar chain lengths and numbers of double bonds, emphasizing the need for reference standards for TGs of interest when determining the regioisomer ratios, as the *sn*‐2 FA cleavage might not always result in DG fragment of the lowest abundance (Baiocchi et al., 2015). Selected TG regioisomers from cocoa butter, palm oil, and certain animal fats have been analyzed (APCI(+)‐MS, [M+H]^+^), but several regioisomer ratios, especially in the animal fats, could not be evaluated due to lack of reference standards or chromatographically overlapping TGs with common structurally informative fragment ions (Fauconnot et al., 2004). In addition to APCI, in‐source fragmentation with atmospheric pressure photoionization (APPI) has been utilized for structural analysis of TGs. While the study was focusing on the TG molecular species composition, determining regioisomer ratios would follow similar principles as with APCI in‐source fragmentation, and the fragment ions resulting from loss of *sn*‐2 FA were less abundant than those from loss of *sn*‐1/3 FA (Abreu et al., 2021).

#### 3.2.3 EIEIO

**Figure 9:**
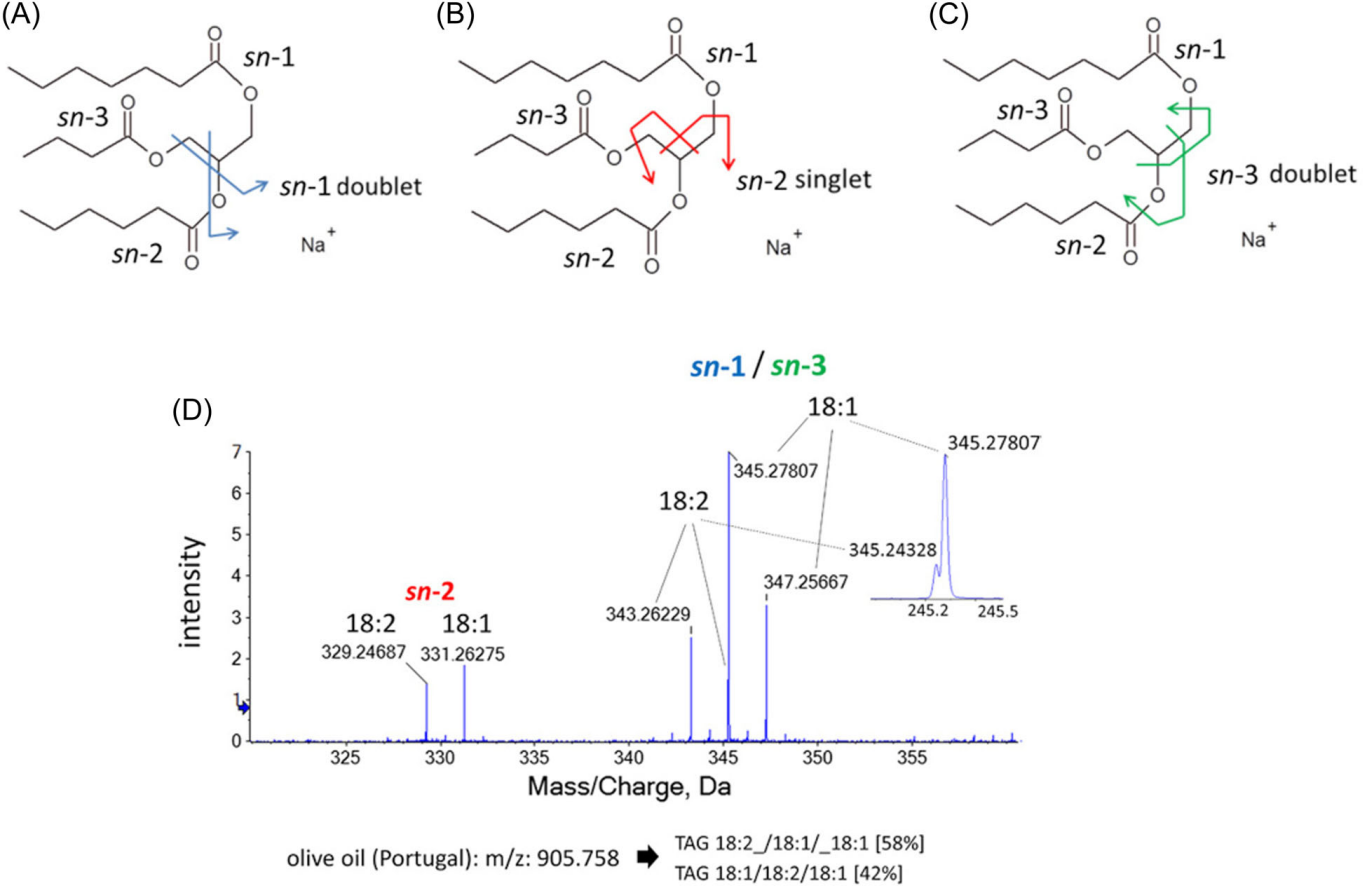
EIEIO cleavage sites of the diagnostic peaks shown in *sn*‐1 doublet (blue) (A), *sn*‐2 singlet (red) (B), and *sn*‐3 doublet (green) (C). EIEIO spectrum of *m/z* 905 precursor ions in olive oil showing an example of deconvolution of TG regioisomers (D). Reprinted from (Baba et al., 2016) with permission of American Society for Biochemistry and Molecular Biology. [Color figure can be viewed at wileyonlinelibrary.com]

EIEIO is a relatively unused technique for structural analysis of TGs, but in recent years it has shown potential for very in‐depth characterization of TG isomers. Unlike CID, which cleaves off the acyl chains, EIEIO breaks the glycerol backbone (Baba et al., 2016), resulting in unique structurally informative fragment ions after loss of two FAs. Cleavage of the glycerol backbone produces two doublet ions (*sn*‐1 and *sn*‐3), both spaced 2 Da apart, and one singlet ion (*sn*‐2) as indicated in Figure 9A–C. Especially useful for identifying TG regioisomers is the formation of *sn*‐2 singlets, which are unique to the FAs attached to *sn*‐2 position. The remaining *sn*‐1/3 FAs can be identified from the two doublet ions, but their *sn*‐positions are indistinguishable. Even though CID heavily favors cleavage of FAs from *sn‐*1/3 positions, it always produces some fragments resulting from *sn*‐2 FA cleavage, which also is influenced by the nature of the FA. However, the uniqueness of the *sn*‐2 singlet from EIEIO fragmentation implies that calibration standards for determining TG regioisomer ratios are of less importance. An example of quantifying regioisomer ratios is shown in Figure 9D, consisting of two *sn*‐2 singlets, identified as FA 18:1 and FA 18:2. Additionally, two *sn*‐1/3 doublets are observed, identified as FA 18:1 and FA 18:2. Based on this information, the regioisomers are identified as TG 18:2_18:1(*sn‐*2)_18:1 and TG 18:1_18:2(*sn‐*2)_18:1, and the regioisomer ratios calculated with the fragment abundances are 58% and 42%, respectively (Baba et al., 2016). The method (ESI(+)‐EIEIO‐MS^2^, [M+Na]^+^) was later expanded and used for profiling TG regioisomers in addition to several other lipid classes in porcine brain (Baba et al., 2018). The acquisition time for each EIEIO spectrum was 1 min. Direct infusion in conjunction with DMS ion mobility separation was utilized. As multiple lipid classes might contain several isobaric species, the DMS separation was used for selecting the class‐specific molecular species. Conventional chromatographic separation would not be viable with such spectra acquisition times (Baba et al., 2018).

#### 3.2.4 Ozone‐induced dissociation (OzID)

Sequential fragmentation with CID followed by OzID has been used for analysis (HESI(+)‐OzID‐CID‐MS^3^, [M+Na]^+^) of TG regioisomers in *Drosophila* flies (Marshall et al., 2016). The used linear ion trap instrument had been modified to seed ozone into the helium buffer gas of the ion trap. After CID of the sodiated [M+Na]^+^ adducts, the resulting DG fragments of interest are trapped in the presence of ozone in the ion trap section, resulting in further fragmentation with higher regioselectivity. While the resulting fragment ion ratios are still influenced by the FAs, the method offers an interesting alternate approach for TG regioisomer analysis (Marshall et al., 2016).

#### 3.2.5 Algorithmic interpretation of MS^*n*^ fragment data

As the fragmentation data of TG regioisomers in natural samples can be complex, and the manual interpretation time consuming and often extremely challenging, several tools have been developed to address this problem. One of the main challenges in the analysis of TG regioisomers in natural samples is the overlapping structurally informative fragment ions from multiple isobaric TG species. Using calibration curves alone might not be enough, as the fragment ion ratios are distorted by interfering fragments from other TGs.

One of the first tools developed to tackle this issue was MSPECTRA (Kurvinen et al., 2001), which utilizes data obtained with a direct inlet shotgun method (CI(–)‐CID‐MS^2^, [M–H]^–^). The software first establishes the TG molecular species composition with the observed FA fragment ion [RCOO]^–^ ratios. These fragments are not regiospecific, but instead they represent the total FA distribution over the fragmented TG species. By performing an optimization algorithm, the software uses the [RCOO]^–^ ion intensities to find combinations of TGs, which result in the least amount of shortage or leftover [RCOO]^–^ ions. The TG regioisomer ratios are then calculated with a similar optimization algorithm utilizing the regioselective [M–H–100–RCOOH]^–^ fragments. One of the limitations of the software is that it effectively uses only one calibration curve for every TG (Kurvinen et al., 2001). The effect of using only one calibration curve was later demonstrated with a wide range of TG regioisomer standards. The calculated results for mixtures of reference standards were mostly accurate, however, there was an undeniable influence on the results caused by an increasing degree of unsaturation in the attached FAs (Fabritius et al., 2020). Considering this, while the accuracy of the calculations could be improved by implementing additional calibration curves, the MSPECTRA software is still a very efficient tool for evaluating relative differences in the TG regioisomer composition among different samples (Fabritius et al., 2020; Kalpio et al., 2021; Kurvinen et al., 2002; Linderborg et al., 2014).

**Figure 10:**
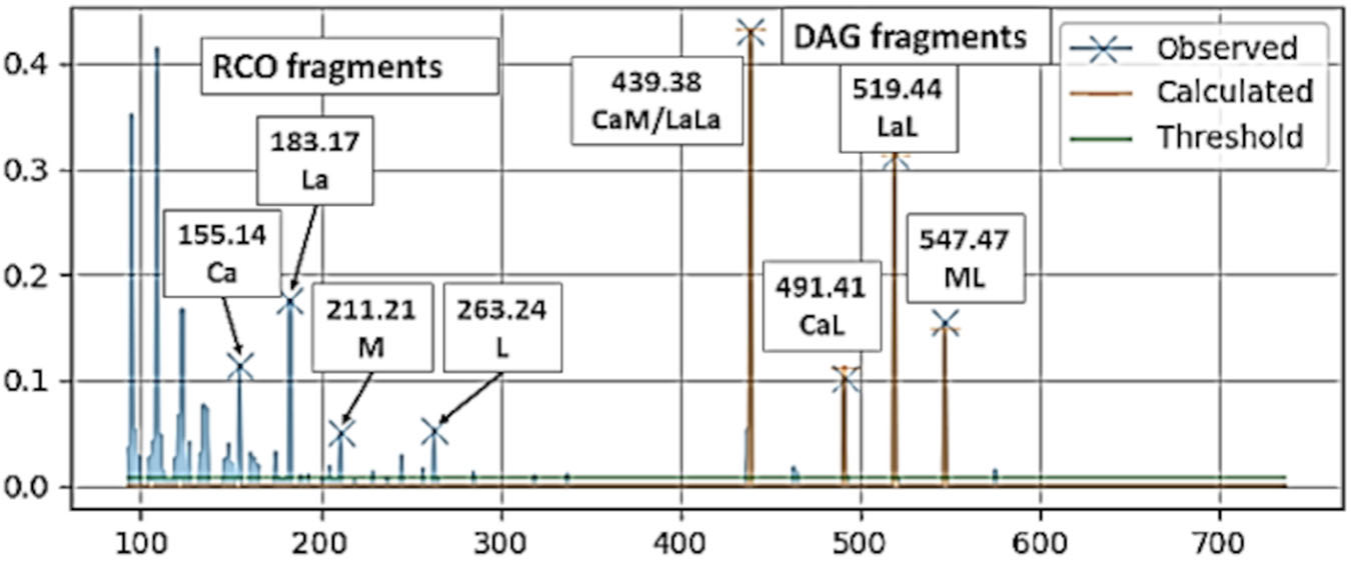
The synthetic DG spectrum of an ACN:DB 42:1 species overlaid on top of the measured fragment ion spectrum. Reprinted with permission from Sazzad et al. (2022), copyright 2022 Elsevier. DG, diacylglycerol. [Color figure can be viewed at wileyonlinelibrary.com]

A similar calculation software (TAG analyzer) has been developed recently (Sazzad et al., 2022). In addition to the optimization algorithm, the software has a built‐in fragmentation model established with TG reference standards. The model takes into account differences in the fragmentation efficiencies of different TG species, including the acyl chain length and the number of double bonds in the *sn*‐2 FA as was observed (ESI(+)‐CID‐MS^2^, [M+NH_4_]^+^) in an earlier study (Tarvainen et al., 2019). The optimization algorithm looks at the fragment spectra and uses the fragmentation model to create a synthetic spectrum to match the observed one as closely as possible (Figure 10). The TG regioisomer abundances used to create the best‐matching synthetic spectra are then reported. The software can accurately identify the most abundant regioisomers within each isobaric TG species, but the accuracy decreases as the relative abundance of the TG molecular species decreases. When the algorithm optimizes the regioisomer abundances, the changes are accentuated in the low abundance TGs, likely resulting in proportionally larger errors (Sazzad et al., 2022). Similar behavior was observed with the MSPECTRA software, where the low abundance TGs within the species produced the highest relative deviations (Fabritius et al., 2020).

An algorithm for analyzing palmitic acid‐containing TG regioisomers has been developed by constructing calibration curves with reference standards (ESI(+)‐CID‐MS^2^, [M+NH_4_]^+^), and subsequently extrapolating fragmentation efficiencies for other TGs. As observed by other authors, the fragmentation was influenced by the double bond number and acyl chain length. Accuracy of the calculation model was demonstrated by comparing the results to manual calculations with the calibration curves. The results for ABC‐type TGs were more in line with the two calculation methods, with a maximum difference of 11%, whereas with the AAB‐type TGs the differences were somewhat higher with a maximum of 27%. As only palmitic acid‐containing TGs were analyzed, more reference standards are needed to expand the method further for a wider range of TGs (Zhang et al., 2022).

Modeling TG fragmentation patterns has been done using other published studies, including data obtained with various different MS instruments using both ESI‐CID‐MS^2^ and APCI‐MS (Balgoma et al., 2019). Fragmentation abundances were instrument and adduct specific. Common trends for fragmentation in each data set were modeled, and the established model was applied to quantify TG regioisomers using their own instrument (ESI(+)‐CID‐MS^2^, [M+NH_4_]^+^) (Balgoma et al., 2019). The results were mostly in agreement with previous studies, including several TGs that were not used in creation of the calculation model. However, the regioisomer ratios of TGs containing two or more highly unsaturated FAs such as FA 20:4, FA 20:5, or FA 22:6 were more difficult to predict. The authors suggested that interlaboratory standardization of ionization and fragmentation conditions could enable comparable analysis of TG regioisomers in high throughput lipidomics with minimal number of standards (Balgoma et al., 2019).

## 4 PL

### 4.1 Separation of PL *sn*‐positional isomers

#### 4.1.1 Chromatographic separation

In contrast to TGs, the chromatographic separation of PL *sn*‐positional isomers remains largely unexplored, and only a handful of examples exist in the literature. Regioisomers of selected PC molecular species, such as PC 16:0_18:2, PC 16:0_20:4, and PC 16:0_22:6, were separated with Acquity C18 BEH (150 × 1 mm, 1.7 μm) RP column (Nakanishi et al., 2010). The regioisomer standards were analyzed as such, and after oxidation as PC hydroxides. The regioisomers of PC 16:0_20:4 and PC 16:0_22:6 were separated in less than 30 min, allowing quantification of regioisomer ratios. PC 16:0_18:2 regioisomers were not separated in the nonoxidized form. Oxidation enhanced the separation, resulting in adequate resolution of PC 16:0_18:2 regioisomers during a 10 min gradient. The relative peak areas of nonoxidized PC regioisomers were approximately the same as the oxidized compounds, suggesting that the ratio of regioisomers is not influenced by oxidation. Therefore, oxidation could be used as a tool to improve chromatographic resolution of PC regioisomers that would not be properly separated in their conventional forms (Nakanishi et al., 2010). Similarly, PC 16:0_20:4 and PC 16:0_22:6 regioisomers were successfully separated using the same Acquity C18 BEH (150 × 1 mm, 1.7 μm) column, but almost no separation was observed for PC 16:0_18:1 regioisomers (Kozlowski et al., 2015b). The regioisomer with a more highly unsaturated FA in the *sn*‐1 position always eluted earlier with the tested PC reference standards. As the degree of unsaturation and the relative differences in the FA acyl chains is reduced, the separation becomes more challenging (Kozlowski et al., 2015b; Nakanishi et al., 2010). Custom capillary columns packed with C18 BEH particles have been used for lipid separations, including some PC regioisomers. Larger differences in the FA chain lengths, such as molecular species PC 2:0_16:0, resulted in baseline separation of the two isomers, while regioisomers of PC 14:0_18:0 were only partially separated (Sorensen et al., 2020). As the examples of chromatographic separation of PL regioisomers are limited and often only C18 columns have been used, it might be worthwhile exploring the capabilities of C30 columns for this purpose as they might provide better selectivity for the regioisomers due to increased hydrophobicity of the stationary phase and interactions with the hydrophobic FA tails of the PLs.

The examples of PL enantiomer (phosphate group in *sn*‐1 or *sn*‐3 position) separation are very limited. It is generally accepted that the phosphate head group in natural PLs is almost exclusively in *sn*‐3 position. However, the experimental evidence supporting this assumption is lacking. Partial separation of racemic dipalmitoyl phosphatidylcholine (palmitic acids in *sn*‐1/2 or *sn*‐2/3 positions and phosphate group in *sn*‐1 or *sn*‐3 position) was achieved using a Chiralpak ID column containing amylose tris(3‐chlorophenylcarbamate) as the chiral selector (Itabashi, 2012). Later, a baseline separation of various PC enantiomers was achieved using a Chiralpak IE column with amylose tris(3,5‐dichlorophenylcarbamate) stationary phase (Kuksis et al., 2016).

#### 4.1.2 Ion mobility

While ion mobility has seen some use for separation of PL regioisomers, most existing applications are only demonstrating the potential with specific compounds, and adaptation to a wider range of regioisomers and PL classes remains a challenge. Utilizing DMS, the effects of adduct type, chemical modifier, and cell temperature were investigated to enhance the separation of molecular species PC 16:0_18:1 regioisomers (Ieritano et al., 2019). Five different adduct types [M+X]^+^ (*X* = H, Ag, Li, Na, or K) were tested. PLs readily form protonated [M+H]^+^ adducts in positive ESI mode, making them an obvious choice for further investigations. However, no regioisomer separation of [M+H]^+^ adducts was observed after seeding the DMS cell with different chemical modifiers such as isopropanol, dichloromethane, acetonitrile, or acetone. [M+Li]^+^ adducts were partially separated, but required high gas pressures, resulting in significant loss of signal intensity. Best separation was achieved with [M+Ag]^+^ adducts and low DMS cell temperature without a chemical modifier in addition to the methanol used as a sample solvent (Ieritano et al., 2019). In another study, [M+Ag]^+^ adducts of PC 16:0_18:1 and 16:0_18:2 regioisomers were also successfully separated using DMS, but regioisomeric separation of the saturated molecular species PC 16:0_18:0 was not achieved (Maccarone et al., 2014). Like silver‐ion chromatography, the amount of double bonds in the attached acyl chains seems to play a critical role in the ion mobility separation of [M+Ag]^+^ adducts (Ieritano et al., 2019; Maccarone et al., 2014).

In a broader study for separation of various PL isomer types with drift tube ion mobility spectrometry (DTIMS), separation of saturated PC regioisomers was also attempted. The utilized adduct ion type was not specified, but only minor differences were observed in the drift times of PC 16:0_18:0 regioisomers, resulting in mostly overlapping peaks and inadequate separation (Kyle et al., 2016). Various adduct types have also been tested with DTIMS, including [M+X]^+^ (*X* = H, Ag, Na, or K) (Groessl et al., 2015). The [M+Ag]^+^ adducts of PC 16:0_18:1 were again the most easily separable ones with highest differences in the CCS values of the two regioisomers. The method was used to quantify PC 16:0_18:1 regioisomer ratios in porcine brain and yeast extract, while no other regioisomer pairs were investigated (Groessl et al., 2015). Partial separation of protonated [M+H]^+^ adducts of PC 16:0_18:1 regioisomers has been achieved using trapped ion mobility spectrometry (TIMS), allowing quantification of regioisomer ratios in human plasma (Fouque et al., 2019).

### 4.2 Identification of PL regioisomers by MS methodologies

There are multiple examples of PL regioisomers analyzed with MS methodologies, but most of them are limited in scope, only focusing on a handful of individual regioisomers. The targeted compounds often consist of PCs only, while other PL classes are mostly neglected. ESI is the most commonly used ionization technique, but also direct surface imaging techniques such as desorption electrospray ionization (DESI) and especially matrix‐assisted laser desorption/ionization (MALDI) are used. Similar to TG regioisomers, when employing MS methodologies the PL regioisomers are typically determined by the fragment ion ratios, which are influenced by the nature of the attached FAs.

#### 4.2.1 CID

**Figure 11:**
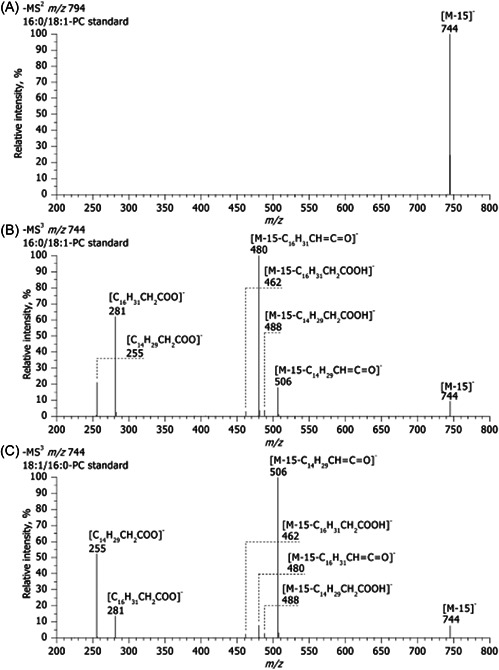
MS^2^ spectrum of a chloride adduct of a PC 16:0/18:1 standard (A) and subsequent MS^3^ spectra of demethylated [M–15]^–^ ions of PC 16:0/18:1 (B) and PC 18:1/16:0 (C). Reprinted with permission from Ekroos et al. (2003), copyright 2003 Elsevier.

A single‐stage fragmentation with CID alone when using positive ionization ESI is often not enough for analysis of PL regioisomers, as the structurally informative fragment ions in most cases are of low abundance (Hsu & Turk, 2009). On negative polarity, sequential CID of chlorinated PC adducts (ESI(–)‐CID‐MS^3^) was first observed to produce high abundance deprotonated FA [RCOO]^–^ and lysophospholipid‐like fragments that can be used for structural assignment of the FA chains (Ekroos et al., 2003). Figure 11A shows the lack of structurally informative fragments after the first CID step, but the sequential fragmentation of the demethylated [M–15]^–^ ions (Figure 11B,C) produces fragments that have reversed abundance ratios for PC 16:0/18:1 and PC 18:1/16:0 regioisomers, allowing identification of regioisomers. Similar methodologies utilizing ammonium adducts have been used to examine PC regioisomers in human plasma (Zacek et al., 2016) and for investigation of the impact of high‐fat diets on PC regioisomer composition in mice (Sundaram et al., 2018). Using ammonium formate as the mobile phase modifier, PCs are ionized as formate adducts [M+HCOO]^–^ that can produce the same structurally informative [RCOO]^–^ and lysophospholipid‐like fragments after a single‐stage CID fragmentation as was demonstrated by the comprehensive work earlier (Hsu & Turk, 2009).

**Figure 12:**
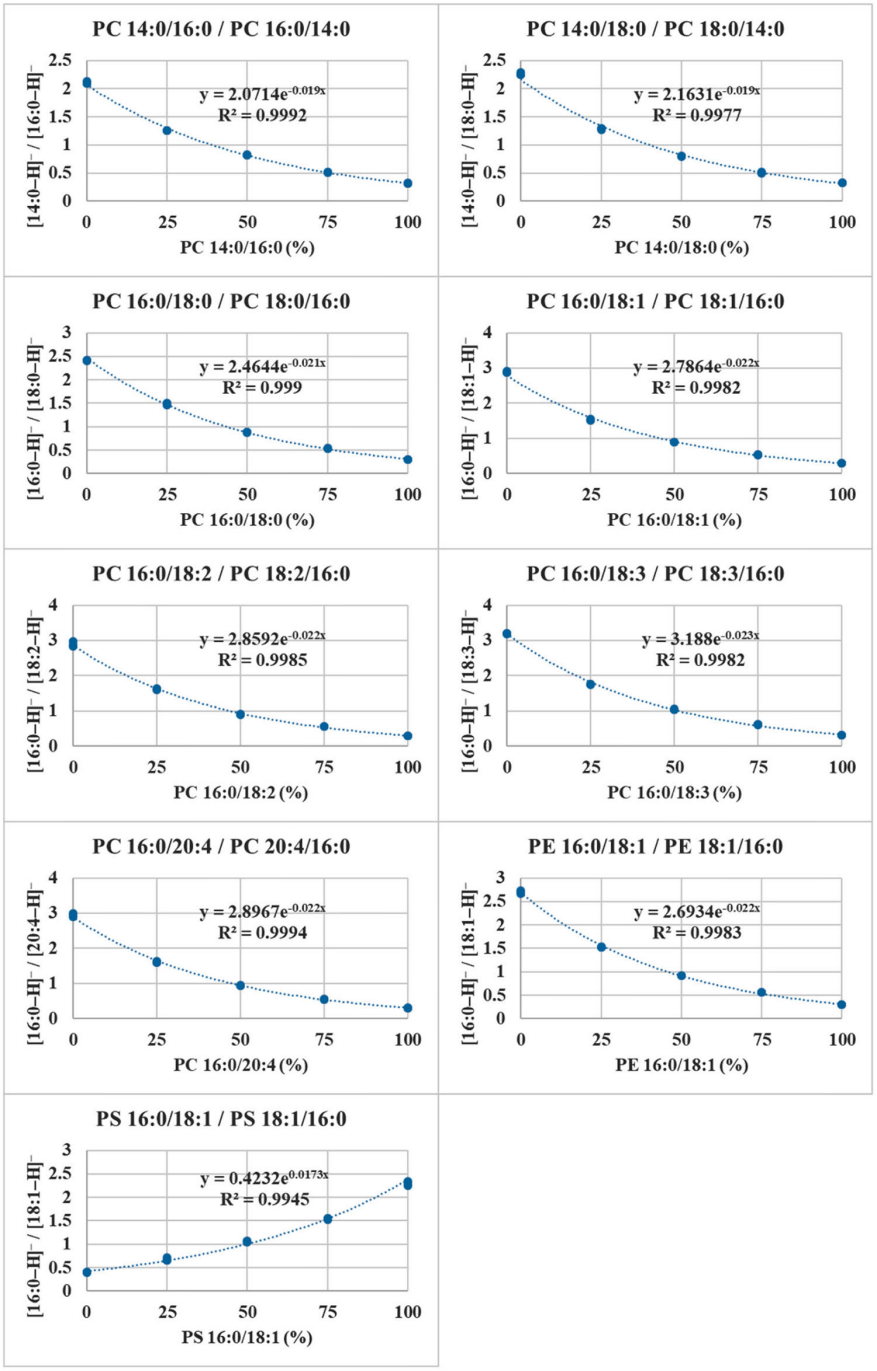
Calibration curves of various regiospecific PL reference standard pairs using [RCOO]^−^ fragment ion ratios. Reprinted with permission from Fabritius and Yang (2021), copyright 2022 John Wiley and Sons. PL, phospholipid. [Color figure can be viewed at wileyonlinelibrary.com]

**Figure 13:**
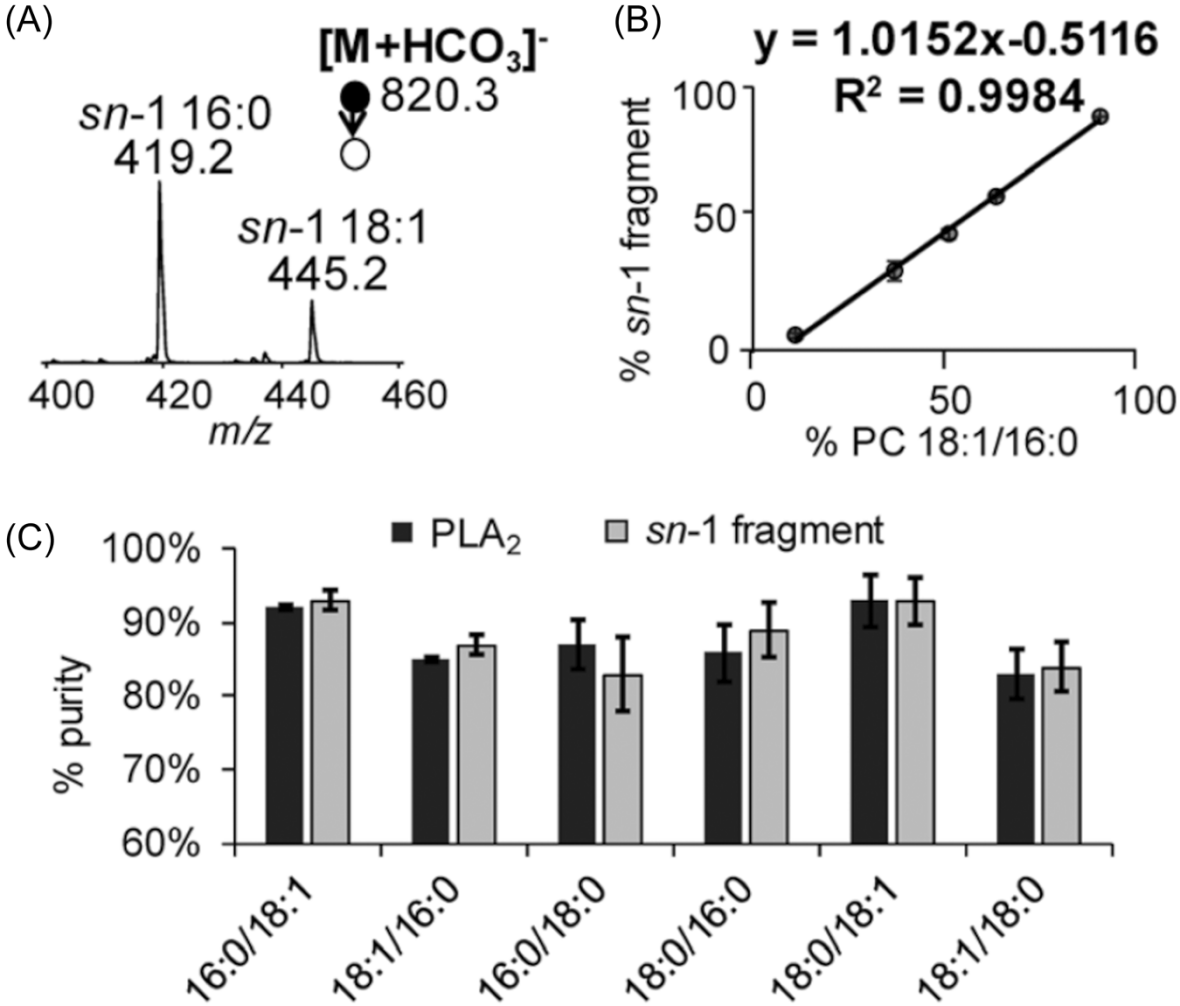
CID spectrum of [M+HCO_3_]^–^ derived from a 2:1 mixture of PC 16:0/18:1 and PC 18:1/16:0 (A), Correlation between *sn*‐1 specific fragment as a function of molar ratio of PC 18:1/16:0 in the reference regioisomer standard mixture (B), Comparisons of the purity of six PC standards measured by the *sn*‐1 fragment and enzymatic PL_2_ method (C). Reprinted with permission from Zhao et al. (2019), copyright 2019 Royal Society of Chemists. PC, phosphatidylcholine.

Several PC calibration curves, one PE and one PS calibration curve were established with reference standards in a recent study, allowing quantification of regioisomer ratios in standard mixtures (Figure 12) (Fabritius & Yang, 2021). The fragmentation of different PCs was influenced by the acyl chain lengths and the numbers of double bonds. Direct infusion of individual regioisomer standards yielded accurate results, but some challenges were encountered during a hydrophilic interaction liquid chromatography (HILIC) analysis of a mixture containing all studied reference compounds. As the HILIC method separated PLs primarily by class, many molecular species within the class are chromatographically overlapping. Due to inadequate precursor ion isolation capabilities of the instrument, regioisomers of molecular species such as PC 16:0_18:2 and PC 16:0_18:3 which only differ in one double bond, were difficult to analyze because interference by fragments from one another, distorting the fragment ion ratios (Fabritius & Yang, 2021). This issue could have been bypassed with better separation of the molecular species, for example using RP chromatography. For PC and PE classes, the fragments produced by cleavage of *sn*‐2 FA from [M+HCOO]^–^ or [M–H]^–^ adducts, respectively, were more abundant than the fragments resulting from *sn*‐1 FA cleavage. The fragmentation pattern for PS was less clear, as the abundance of FA fragments was [R_1_COO]^–^ > [R_2_COO]^–^, but for the lysophospholipid‐like fragments the abundance ratio was reversed [M–H–87–R_2_COOH]^–^ > [M–H–87–R_1_COOH]^–^. This discrepancy could be explained by more complex fragmentation mechanisms as was theorized earlier (Hsu & Turk, 2005; Hvattum et al., 1998). Nevertheless, a calibration curve was created with regiopure reference standards, and the fragment ion ratios were successfully used to determine regioisomers of PS 16:0_18:1 molecular species in a standard mixture. The use of both [RCOO]^–^ and the lysophospholipid‐like fragments for regioisomer calculations of PC, PE, and PS classes was demonstrated (Fabritius & Yang, 2021). Using CID on negative polarity, PCs are commonly analyzed as formate [M+HCOO]^–^ adducts. However, utilizing ammonium bicarbonate resulted in significantly higher sensitivity of the precursor ions (nanoESI(–)‐CID‐MS^2^), forming [M+HCO_3_]^–^ adducts instead (Zhao et al., 2019). Additionally, CID of the [M+HCO_3_]^–^ adducts produced fragments with *sn*‐1 FA connected to a dehydro‐glycerol backbone with the *sn*‐3 hydroxyl esterified by ethyl phosphate. This fragment was *sn*‐1 specific, allowing analysis of PC regioisomers utilizing the *sn*‐1 fragment ion ratios and calibration curves with standards (Figure 13). The developed method was used to investigate certain PC regioisomer ratios in human breast cancer tissue (Zhao et al., 2019).

A combination of the FA fragment [RCOO]^–^ ion ratios and partial chromatographic separation has been used in calculating regioisomer ratios (Wozny et al., 2019). As the regioisomers such as PC 16:0_18:1 and PC 18:1_16:0 had only very minor difference in the retention time with the used C18 RP column, the fragment ion ratios in the leading edge, center and the tailing edge of the peak were also slightly different. These minor differences of individual regioisomer standards were used to create elution profiles that were used to monitor [R_2_COO]^–^ > [R_1_COO]^–^ fragment ion ratios at specific retention times. The method was used to quantify regioisomers of PC 16:0_18:1 and PC 18:0_18:1 in bovine liver and *Escherichia coli* lipid extracts (Wozny et al., 2019).

In positive ionization mode, sequential fragmentation with CID has been used (nanoESI(+)‐CID‐MS^3^) to produce structurally informative fragments after Paterno‐Buchi (PB) photochemical derivatization (Cao et al., 2020). After the loss of PC or PE head group in the first step, the resulting fragments were further fragmented, yielding *sn*‐1 and *sn*‐2‐specific fragments. A total of seven different PB reagents were screened for highest abundance of *sn*‐specific fragment ions and 2‐acetylpyridine was selected. The regioisomer ratios within one species were calculated by dividing the *sn*‐specific ion intensity of one isomer by intensities of all *sn*‐specific ions within the species. The method was used for studying differences in PC regioisomer compositions of human lung cancer tissue, breast cancer cell lines, and human plasma from diabetic patients (Cao et al., 2020). A similar method utilizing PB derivatization with 2‐acetylpyridine and sequential CID was recently used for investigating (HESI(+)‐CID‐MS^3^) PC regioisomers in bovine milk. The focus was on studying fragmentation behavior of different PC molecular species. Accurate quantification of regioisomer ratios was not attempted in this study, and it was noted that calibration curves would be needed for such analysis (Liu & Rochfort, 2022). Utilizing electrochemical reactions to form [M+Co]^2+^ adduct ions, PC regioisomers were studied (nanoESI(+)‐CID‐MS^2^) after fragmentation with CID (Tang et al., 2022). The nanoESI ion source was fitted with metallic cobalt wire, leading to anodic corrosion during the analysis and formation of the doubly charged cobalt adducts. Other metallic complexing agents such as Cu, Ag, Au, and Fe were also tested, but the fragmentation patterns were not informative for *sn*‐positional analysis. The PC 16:0_18:1 regioisomer ratios were calculated with a calibration curve using [R_2_COO+Co]^+^ and [M+H–R_2_COOH]^+^ fragments. Calculating accurate regioisomer ratios for other molecular species would have required additional calibration standards, and the method was used for monitoring relative changes in the PC regioisomers of mouse prostate cancer tissue (Tang et al., 2022).

Surface imaging with three CID fragmentation steps was used (nanoDESI(+)‐CID‐MS^4^) in analysis of silver adducts [M+Ag]^+^ of PCs (Lillja & Lanekoff, 2022). The resulting fragments of PC 16:0_18:1 in MS^4^ were mostly *sn*‐2 specific, with a minor abundance of *sn*‐1 specific fragments. The method was used for imaging regioisomers of molecular species such as PC 16:0_18:1, PC 18:0_18:1, and PC 16:0_20:1 in different regions of mouse brain. In addition to regioisomer ratios, also quantification of the PL regioisomer abundances was performed with this method (Lillja & Lanekoff, 2022). MALDI with a single‐stage CID has been used with a charge inversion technique, changing the initial positively charged adducts to negative using sequential ESI following MALDI (MALDI(+)/ESI(–)‐CID‐MS^2^). The PCs are initially ionized as [M+H]^+^, but utilizing 1,4‐phenylenedipropionic acid (PDPA) and negative polarity ESI, complex [M+PDPA–H]^–^ and demethylated [M–CH_3_]^–^ ions are formed. The [M–CH_3_]^–^ ions were further fragmented with CID, yielding [RCOO]^–^ fragments and allowing regioisomer analysis of PCs utilizing calibration curves. The initial ionization on positive polarity allows for higher sensitivity, while the charge inversion to negative enables structural characterization (Randolph et al., 2020).

#### 4.2.2 OzID

**Figure 14:**
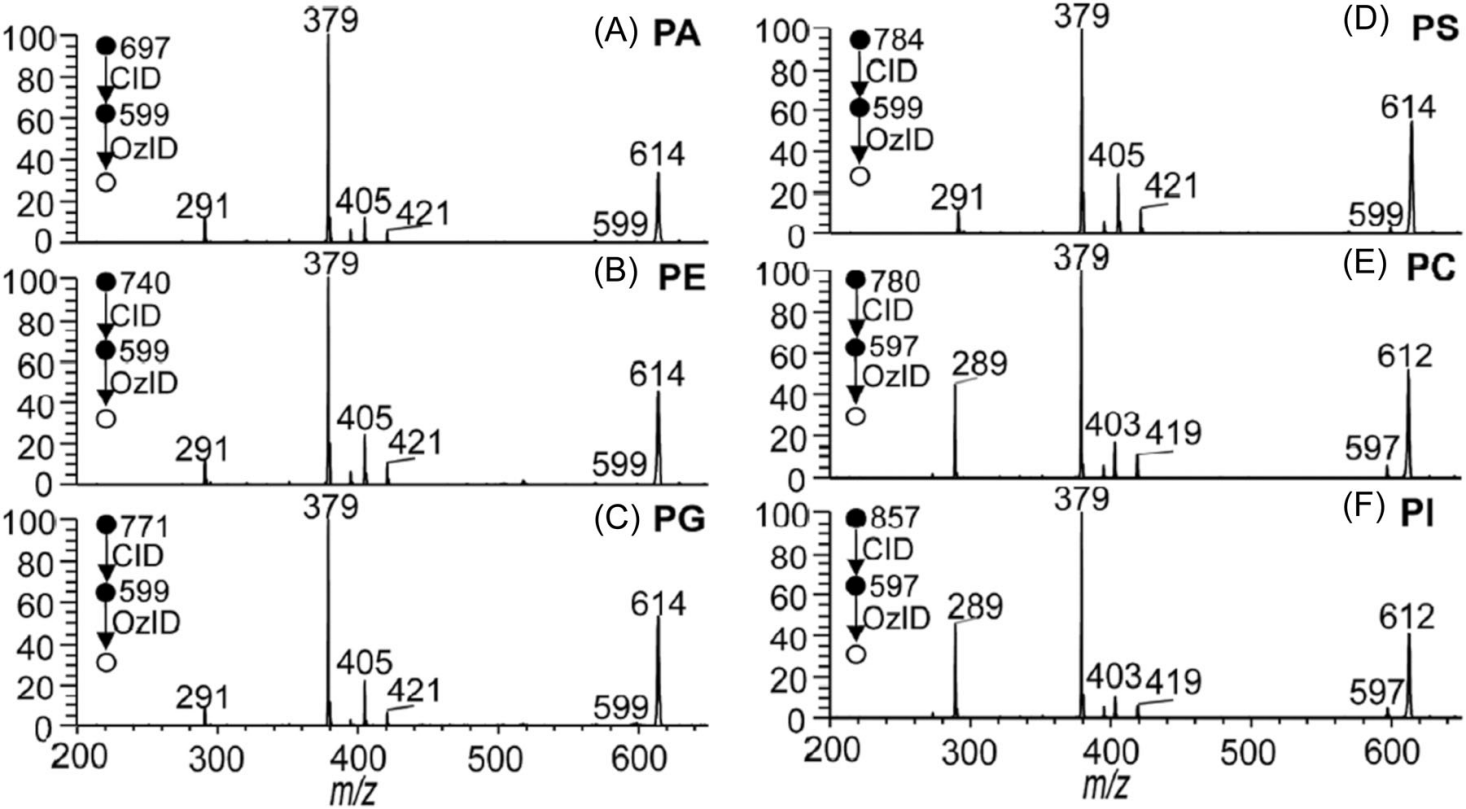
CID‐OzID on the ions corresponding to complete phospholipid headgroup losses from precursors ions PA 16:0/18:1 (A), PE 16:0/18:1 (B), PG 16:0/18:1 (C), PS 16:0/18:1 (D), PC 16:0/18:2 (E), and PI 16:0/18:2 (F). Reprinted with permission from Pham et al. (2014), copyright 2014 Royal Society of Chemists.

OzID is commonly used for identifying the double bond locations on the FA moieties, but it can also be utilized in PL regioisomer analysis, often in tandem with CID (CID‐OzID). Chromatographic separation is rarely utilized with CID‐OzID, instead, the commonly used methods include direct infusion with ESI (Batarseh et al., 2018; Pham et al., 2014) or MS imaging (Claes et al., 2021; Kozlowski et al., 2015a; Paine et al., 2018; Young et al., 2022) on positive polarity with sodiated [M+Na]^+^ adducts. Applicability of CID‐OzID for PL regioisomer analysis was demonstrated (ESI(+)‐CID‐OzID‐MS^3^) with reference standards of six different classes (PA 16:0/18:1, PC16:0/18:2, PE 16:0/18:1, PG 16:0/18:1, PI 16:0/18:2, and PS 16:0/18:1) (Pham et al., 2014). After loss of the head group in CID, the resulting ion was further fragmented with OzID. The CID‐OzID fragment profiles of the six different classes (Figure 14) look remarkably similar (Pham et al., 2014), unlike with CID only where the PL class significantly influences the fragmentation pattern (Fabritius & Yang, 2021). This indicates that the removal of the head group in the first fragmentation step with CID followed by OzID could make analysis of PL classes such as PI or PS more approachable if the more readily available reference standards like PC or PE could be utilized to establish calibration curves.

A triple‐stage fragmentation (MALDI(+)‐OzID‐CID‐OzID‐MS^4^) has been used for full structural characterization of certain PCs, including regioisomers and double bond locations (Young et al., 2022). In the first stage, the [M+Na]^+^ precursor was subjected OzID, followed by selection of one aldehyde product ion of one DB position for CID fragmentation. After the loss of the head group, the final OzID stage produced fragments that enabled regioisomer analysis. PC regioisomers in prostate cancer tissue were analyzed (Young et al., 2022).

The ozonolysis for CID‐OzID fragmentations can be performed for example in the trapping region of an ion trap instrument (Paine et al., 2018) or in the ion mobility region of an IMS instrument (Claes et al., 2021). Depending on the instrument type, the reaction rate for the ozonolysis can be a limiting factor with MS imaging if high enough ozone concentrations cannot be achieved. Significantly faster reaction rates resulting in shorter analysis times were observed when OzID was implemented in the high‐pressure region of an ion mobility instrument (Claes et al., 2021). Both methods (MALDI(+)‐CID‐OzID‐MS^3^) were used to investigate PC regioisomers in different regions of rat brain (Claes et al., 2021; Paine et al., 2018). In addition to MS imaging with MALDI, also DESI has been used in conjunction with CID‐OzID fragmentation for analysis of select PC regioisomers in various animal tissues and chicken egg yolk (Kozlowski et al., 2015a).

#### 4.2.3 Other dissociation techniques

In‐source fragmentation with MALDI on positive polarity has been used for PL regioisomer analysis without a dedicated fragmentation step (Wang & Hsu, 2022). In‐source fragmentation was generated by increased laser irradiation, resulting in structurally informative fragments after a preferential loss of *sn‐*2 FA, similar to those observed with CID. While capable of resolving PL individual regioisomers, the method requires an efficient separation method for structural characterization of PLs in complex natural samples (Wang & Hsu, 2022).

EID, including EIEIO of PLs produce a much wider variety of structural fragments compared with CID, which also allows differentiation of double bond isomers in addition to regioisomers (MALDI(+)‐EID‐MS^2^) (Jones et al., 2015). A variety of different fragments obtained with EID could be used for studying PC 16:0_18:1 and PC 18:0_18:1 regioisomers in different rat brain regions utilizing calibration curves. *Sn*‐specific fragments resulting from a cleavage along the glycerol backbone were also observed (MALDI(+)‐EID‐MS^2^), but the intensities were low, limiting their usefulness in determining regioisomer ratios (Born & Prentice, 2020). The *sn*‐specific fragments were more prominent in another study (ESI(+)‐EIEIO‐MS^2^), where PC regioisomers were calculated using *sn*‐1‐specific ion ratios resulting from C_1_ to C_2_ carbon bond cleavage on the glycerol backbone (Campbell & Baba, 2015). When analyzing a PC 16:0/18:1 standard, a small amount of *sn*‐2‐specific fragment resulting from loss of FA 16:0 was observed. This was attributed to isomeric impurity of the standard, as the other regioisomer standard PC 18:1/16:0 produced only appropriate *sn*‐1‐specific 18:1 and *sn*‐2‐specific 16:0 fragments (Campbell & Baba, 2015). The method (ESI(+)‐EIEIO‐MS^2^) was later used to analyse various other lipids, including regioisomers of PC, PE, PI, and PS in porcine brain. The regioisomer ratios seem to have been calculated simply based on the *sn*‐specific fragment ratios (Baba et al., 2018).

A comparison between CID, higher‐energy collisional dissociation (HCD), and ultraviolet photodissociation (UVPD) on the structural fragments of PC standards has been performed (HESI(+)‐CID/HCD/UVPD‐MS^2^) utilizing metal ion complexes as the precursor ions (Becher et al., 2018). A total of 11 different metal salts were screened for best suitability with UVPD, of which FeCl_2_ forming [M+Fe]^2+^ ions was selected due to the highest abundances of structurally informative fragments. Regardless of the higher *sn*‐specificity of the UVPD fragments compared to those obtained with CID or HCD, calibration curves with reference standards are still required for accurate analysis of regioisomer ratios. UVPD fragmentation was used for analysis of PC regioisomers in mouse pancreas lipid extract (Becher et al., 2018). HCD has also been used in tandem with UVPD, resulting in loss of PL head group of the [M+Na]^+^ precursors in the first fragmentation step and formation of structurally informative fragments in the second step (nanoESI(+)‐HCD‐UVPD‐MS^3^) (Williams et al., 2017). Similar to CID‐OzID fragmentation of different PL classes (Pham et al., 2014), CID/HCD‐UVPD also produced nearly identical fragment spectra for species with the same FA composition such as PA 16:0/18:1, PC 16:0/18:1, PE 16:0/18:1, PG 16:0/18:1, PI 16:0/18:1, and PS 16:0/18:1 after the initial head group loss (Williams et al., 2017).

## 5 CONCLUSIONS

Chromatography can offer targeted approaches for *sn*‐positional isomers of specific TGs and PLs species of interest, but the methods are often very time‐consuming and not suitable for untargeted analysis of a wide spectrum of isomers. Various methods have been investigated for analysis of TG and PL regioisomers based on tandem mass spectrometry in shotgun mode or combined with liquid chromatography. Ion mobility for separation of regioisomers is an emerging technique and still quite unused, and the full potential remains likely to be explored. CID is the most frequently used fragmentation technique for regioisomeric analysis of both TGs and PLs taking advantage of differential dissociation energy of FA from the primary and secondary positions. CID in tandem with OzID has shown potential to differentiate double‐bond isomers in addition to regioisomers. Determining the regioisomer ratios using MS methods usually requires calibration curves based on the structurally informative fragment ion ratios. Adapting the developed MS methods for use in another laboratories is not straightforward, as the fragment ion ratios can also be influenced by instrument and collision gas type. With TGs there is also the challenge of isobaric fragments resulting from multiple chromatographically overlapping molecular species distorting the fragment ion spectra. Calculating TG regioisomers from convoluted spectra with calibration curves alone is not viable, and automated optimization algorithms has been shown to offer useful solutions in such cases.

Regiopure standards can be expensive and often outright not available for certain PL classes. While most fragmentation methods produce fragments related to FA cleavage at different ratios from both *sn*‐1 and *sn*‐2 positions of PL, certain methods such as EIEIO can produce unique *sn*‐specific fragments. However, the structural features of FA, that is, the chain lengths as well as the number and position of double bonds of FAs likely influence the efficiency of *sn*‐specific fragmentation; therefore, it is possible that calibration curves with different FA combinations are necessary for accurate quantification of regioisomers. Establishing fragmentation models using commercially available standards can help in properly investigating regioisomer abundances of complex samples, reducing the need for a much wider variety of reference standards. This has been done for TGs to assist calculations, and similar approach could be utilized for PL regioisomers as well.

In conclusion, since an acceptable chromatographic separation of isobaric TG molecular species is often not achievable, careful assessment of the effects of interfering isobaric fragments is necessary when calculating regioisomer ratios for each fragment ion spectrum. Individual calibration curves are insufficient for complex natural samples, as isobaric DG fragments could originate from various TG species. A more effective approach involves using a general fragmentation model combined with an optimization algorithm. This method recreates the observed spectrum by calculating the regioisomer abundances responsible for the original spectrum, showing promise for untargeted TG regioisomer analysis and mitigating the effects of isobaric fragments. The algorithmic tool *TAG Analyzer*, which was recently developed in our lab using a fragmentation model based on a wide range of standard curves, has been proven to be effective in resolving the comprehensive TG regioisomer profile of the most complex natural fats and oils such as human milk.

With PLs the interference of isobaric fragment ions is of less importance, but lack of reference standards for various PL classes hinders development of fragmentation models. Future research should be directed to further development and integration of untargeted regioisomer analysis methods into standard lipidomics protocols to explore regioisomeric profiles of glycerolipids in tissues as biomarkers of health and diseases.
